# Unexpected formation of polymeric silver(I) complexes of azine-type ligand via self-assembly of Ag-salts with isatin oxamohydrazide

**DOI:** 10.1098/rsos.180434

**Published:** 2018-07-25

**Authors:** Saied M. Soliman, Jörg H. Albering, Assem Barakat

**Affiliations:** 1 Department of Chemistry, Faculty of Science, Alexandria University, PO Box 426 Ibrahimia, 21321 Alexandria, Egypt; 2 Department of Chemistry, Rabigh College of Science and Art, King Abdulaziz University, Jeddah, Saudi Arabia; 3 Graz University of Technology, Mandellstrasse 11 (III), A-8010 Graz, Austria; 4 Department of Chemistry, College of Science, King Saud University, PO Box 2455, Riyadh 11451, Saudi Arabia

**Keywords:** isatin oxamohydrazide, azine, polymeric silver(I) complexes, topology analysis

## Abstract

Isatin oxamohydrazide (**L**) reacted with the aqueous solution of silver nitrate at room temperature afforded the polymeric silver(I) nitrato complex, [Ag_2_L′(NO_3_)_2_]*_n_*, **(1)** of the azine ligand (**L′**). Similarly, the reaction of **L** with silver(I) perchlorate gave the [Ag_2_L′_2_(ClO_4_)_2_]*_n_*, **(2)** coordination polymer. Careful inspection of the crystals from the nitrato complex preparation showed the presence of another crystalline product which is found to be [Ag(Isatin-3-hydrazone)NO_3_], **(3)** suggesting that the reaction between silver(I) nitrate and **L** proceeds first by the hydrolysis of **L** to the isatin hydrazone which attacks another molecule of **L** to afford **L′**. Testing metal salts such as Ni^2+^, Co^2+^, Mn^2+^, Cu^2+^ and Cd^2+^ did not undergo any reaction with **L** either under the same reaction conditions or with heating under reflux up to 24 h. Treatment of the warm alcoholic solution of **L** with few drops of 1 : 1 (v/v) hydrochloric acid gave the free ligand (**L′**) in good yield. The [Ag_2_L′(NO_3_)_2_]*_n_* complex forms a two-dimensional infinite coordination polymer, while the [Ag_2_L′_2_(ClO_4_)_2_]*_n_* forms one-dimensional infinite chains with an alternating silver-azine backbone. Quantitative analysis of the intermolecular interactions in their crystals is made using Hirshfeld surface analysis. Density functional theory studies were performed to investigate the coordination bonding in the studied complexes.

## 1. Introduction

Silver and silver compounds attract great interest from researchers for many reasons. They have interesting antimicrobial actions [1–4]. The antibiotic action of silver is 1000 times more effective [5,6] than that of the traditional antibiotics. Hence, it could present a promising solution to overcome the problem of antibiotic-resistant bacteria [7–10]. Recently, silver compounds with anti-tumour activity have been described in the literature [11]. Also, Ag(I) compounds have interesting application as catalysts for organic transformation reactions [12,13]. Structurally, a d^10^ metal ion such as Ag^+^ can form many coordination geometries with interesting molecular and supramolecular architectures [14–19].

**Scheme 1.**
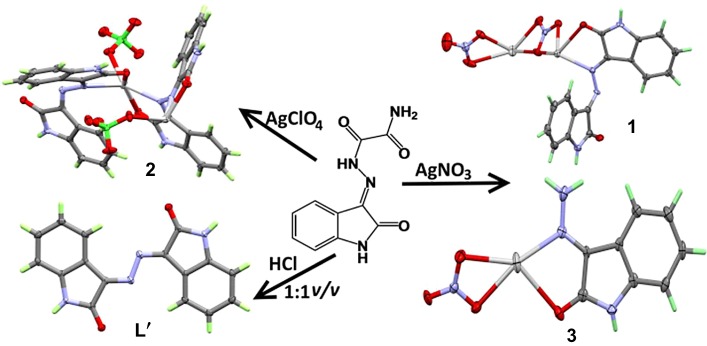
Synthesis of the ligand **L′** and its silver(I) complexes.

On the other hand, oxamohydrazides are well-known powerful ligands according to the literature [20–23]. Radanović *et al.* [24] reported that no complexation occurred when these ligands were treated with Cr(III) salts; instead, the azine product is obtained by an electrophilic acyl substitution catalysed by Cr(III). Here, we report, for the first time, the silver(I) catalysed hydrolytic reaction of the oxamohydrazide **L** to the corresponding azine ligand **L′** and *in situ* complexation with Ag(I) via self- assembly (scheme 1). A plausible number of Ag(I) complexes were synthesized and well characterized. The reactions form polymeric silver(I) complexes with the azine-type ligand (**L′**) at room temperature in the water/methanol mixture. Trial to synthesize the ligand (**L′**) is succeeded by the direct reaction of the hot alcoholic solution of oxamohydrazide (**L**) in the acidic medium (1 : 1 v/v HCl), under reflux for 1 h. The molecular structures of the complexes and the ligand (**L′**) were determined by the X-ray single crystal structure and further examined using Hirshfeld topology analysis. Additionally, the Ag–N and Ag–O coordination interactions were investigated in the framework of atom in molecules (AIM) and natural bond orbital (NBO) analysis.

## 2. Experimental set-up

### 2.1. Material and instrumentation

All chemicals were purchased from Aldrich Company and were used without further purification. Elemental analysis was carried out using Perkin-Elmer 2400 Elemental Analyzer; CHN mode. JEOL-400 NMR spectrometer was used for recording NMR spectra in DMSO-*d*_6_.

### 2.2. Synthesis of isatin oxamohydrazide; **L**

To a solution of isatin (10 mmol, 1.47 g), a drop of acetic acid and oxamic acid hydrazide (10 mmol, 1.03 g) in EtOH (20 ml) were mixed and heated under reflux for 3 h (TLC 20% EtOAc/*n*-hexane). The product was precipitated and the reaction mixture was cooled down and filtered off then washed with a small amount of EtOH to afford the final product (**L**).

Yield; **C_10_H_8_O_3_N_4_** (**L**) **90%:** Anal. calculated: C, 51.73; H, 3.47; N, 24.13; found: C, 51.72; H, 3.47; N, 24.14. ^1^H-NMR for oxamohydrazide (400 MHz, DMSO-*d*_6_) *δ*: 14.02 (s, 1H, N**H**), 11.29 (s, 1H, N**H**), 8.53 (s, 1H, N**H**), 8.17 (s, 1H, N**H**), 7.58 (d, 1H, Ar-**H**), 7.39 (t, 1H, Ar-**H**), 7.08 (t, 1H, Ar-**H**), 6.92(d, 1H, Ar-**H**); ^13^C-NMR (100 MHz, DMSO-*d*_6_) *δ*: 162.5, 160.9, 157.2, 143.1, 140.3, 132.5, 122.6, 121.5, 119.6, 111.0 (electronic supplementary material, figure S1).

### 2.3. Synthesis of the Ag(I)-complexes

Aqueous solution of silver(I) nitrate or perchlorate (1 mmol) was added to the methanolic solution of the ligand (**L**, 1 mmol) at room temperature. The solution was filtered and after one week, dark red crystals of [Ag_2_L′(NO_3_)_2_]_n_ **(1)** and [Ag_2_L′_2_(ClO_4_)_2_]_n_ **(2)** were obtained. Careful inspection of the crystals from the nitrato complex preparation showed that another crystalline form has orange-brown colour. The crystals are identified using X-ray measurements to be [Ag(Isatin-3-hydrazone)NO_3_] **(3)**. Interestingly, the reaction of the ligand (**L**) with different divalent metal ions (Zn, Co, Ni, Cu and Cd) did not afford any reaction using the same reaction route and even by reflux up to 24 h. On the other hand, after the treatment of the warm ligand (**L**) solution in methanol with few drops of hydrochloric acid (1 : 1 v/v), the solution turns dark red. The mixture is heated under reflux for 1 h; then, the filtered solution is left to crystallize. After 1 day, red coloured crystals were collected from the solution.

Yield; **C_16_H_10_Ag_2_N_6_O_8_; (1) 75%:** Anal. calculated: C, 30.50; H, 1.60; N, 13.34; found: C, 30.49; H, 1.59; N, 13.34. IR (KBr, cm^−1^): 3284, 1720, 1606, 1384, 1342. ^1^H-NMR (400 MHz, DMSO-*d*_6_) *δ*: 11.27 (s, 2H, N**H**), 7.57 (d, 2H, Ar-**H**), 7.38 (t, 2H, Ar-**H**), 7.07 (t, 2H, Ar-**H**), 6.92 (d, 2H, Ar-**H**) (electronic supplementary material, figure S1).

Yield; **C_32_H_20_Ag_2_Cl_2_N_8_O_12_; (2) 72%:** Anal. calculated: C, 38.62; H, 2.03; N, 11.26; found: C, 38.61; H, 2.03; N, 11.25. IR (KBr, cm^−1^): 3270, 1730, 1717, 1702, 1614, 1099, 923, 881. ^1^H-NMR (400 MHz, DMSO-*d*_6_) *δ*: 10.99 (s, 2H, N**H**), 7.50 (d, 2H, Ar-**H**), 7.41 (t, 2H, Ar-**H**), 7.00 (t, 2H, Ar-**H**), 6.91 (d, 2H, Ar-**H**) (electronic supplementary material, figure S1).

Yield; **C_8_H_7_AgN_4_O_4_; (3):** Anal. calculated: C, 29.03; H, 2.13; N, 16.92; found: C, 29.04; H, 2.14; N, 16.92. IR (KBr, cm^−1^): 3445, 3281, 1717, 1680, 1604, 1505, 1384, 1356. ^1^H-NMR (400 MHz, DMSO-*d*_6_) *δ*: 10.53 (s, 1H, N**H**), 8.81(s, 2H, N**H_2_**); 7.92 (d, 1H, Ar-**H**), 7.24 (t, 1H, Ar-**H**), 7.00 (t, 1H, Ar-**H**), 6.87 (d, 1H, Ar-**H**) (electronic supplementary material, figure S1).

Yield; **C_16_H_10_N_4_O_2_ (L′) 89%:** Anal. calculated: C, 66.20; H, 3.47; N, 19.30; found C, 66.21; H, 3.47; N, 19.29. IR (KBr, cm^−1^): 3277, 1702, 1614, 1591. ^1^H-NMR (400 MHz, DMSO-*d*_6_) *δ*: 10.99 (s, 2H, N**H**), 7.51 (d, 2H, Ar-**H**), 7.42 (t, 2H, Ar-**H**), 7.01 (t, 2H, Ar-**H**), 6.91 (d, 2H, Ar-**H**) (electronic supplementary material, figure S1).

### 2.4. X-ray measurements

The crystallographic data for the compounds **[Ag_2_L′(NO_3_)_2_]*_n_***, **[Ag_2_L′_2_(ClO_4_)_2_]*_n_*** and **[Ag(Isatin-3-hydrazone)(NO_3_)]** as well as for the free ligand **L′** were collected using a Bruker D8 Quest diffractometer with graphite monochromated Mo K*α* radiation and a photon detector. Absorption corrections were performed by SADABS [25]. The structures were solved by direct methods and all non-hydrogen atoms were localized on difference Fourier maps and refined in subsequent full-matrix least-squares calculations including anisotropic atomic displacement parameters. The hydrogen atoms bonded to carbon were refined with the riding model implemented in SHELX. H atoms bonded to the nitrogen atoms could be detected properly and were refined using a DFIX command in SHELX. All calculations were performed using the Bruker APEX III program system and the SHELXTL program package [26]. The crystallographic data, criteria for the intensity data collection, and some features of the single crystal structure refinements are listed in electronic supplementary material, table S1.

### 2.5. Hirshfeld surface analysis

Crystal Explorer 3.1 program [27] was used to analyse quantitatively the different intermolecular interactions in the studied silver complexes [28] as well as the possibility of π–π stacking among molecular units.

### 2.6. Computational details

Density functional theory (DFT) calculations were carried out using Gaussian 09 software package [29]. The X-ray structure coordinates were used to perform single point calculations using three DFT methods. The Becke three-parameter Lee–Yang–Parr (B3LYP) [30–33], WB97XD, which includes empirical dispersion [34], and the long range-corrected version of wPBE [35] were used. The cc-PVTZ basis sets are used for non-metal atoms and cc-PVTZ-PP for silver is used [36,37] (https://bse.pnl.gov/bse/portal). Multwfn [38] program was used to compute the AIM topological parameters [39]. The NBO 3.1 [40] program implemented in Gaussian 09 software is used for NBO calculations.

## 3. Results and discussion

### 3.1. Synthesis

**Scheme 2.**
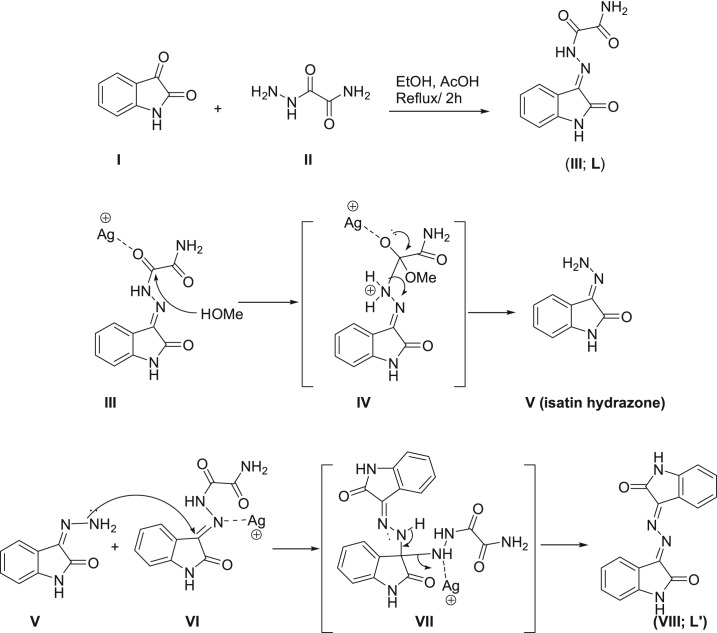
Synthesis of the isatin oxamohydrazide, **L** (upper) and the probable mechanism of Ag^+^-catalysed formation of **L′** (lower).

The role of silver ion was to increase the electrophilicity of carbonyl hydrazide carbon (scheme 2) and/or to increase the acidity of methanol, so that the hydrazide group was hydrolysed and the resulting hydrazino group could subsequently attack hydrazone carbon in another oxamohydrazide molecule in which Ag(I) might also increase its electrophilicity, resulting in the azine product. Finally, the silver complexes were obtained. In the presence of acidic medium, the free ligand **L′** is obtained suggesting the same role of the hydronium ion as silver ion to catalyse its formation.

### 3.2. X-ray structure description

#### 3.2.1. Crystal structure of [Ag_2_L′(NO_3_)_2_]*_n_*; **1**

**Figure 1.**
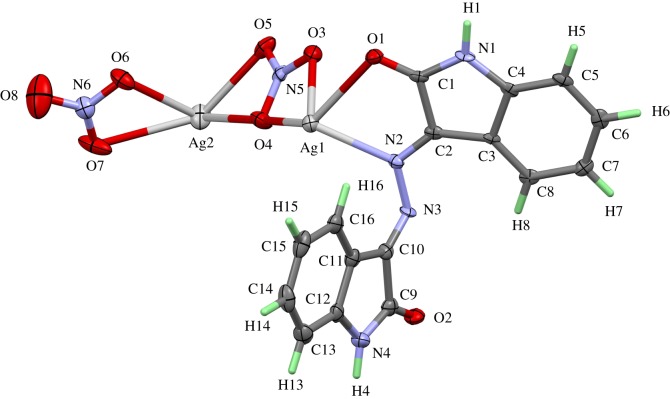
Numbering scheme and atomic displacement ellipsoids drawn at 50% probability level for the asymmetric unit of complex **1**.

The [Ag_2_L′(NO_3_)_2_]*_n_* complex crystallizes in the centrosymmetric space group *P*2_1_/*c* with one formula unit per asymmetric unit and *Z* = 4. The numbering scheme of this complex is shown in figure 1. The crystal structure contains two differently coordinated silver sites. Ag(1) is coordinated by two oxygen atoms belonging to a nitrate ion (O(3) and O(4)) acting as a bidentate ligand, two oxygen atoms of the carbonyl groups (O(1) and O(2)) of the ligand molecule and the two nitrogen atoms N(2) and N(3) of the azine group. The atoms O(1) and N(2) belong to the same coordinating **L′** molecule, while O(2) and N(3) are part of a neighbouring ligand. The Ag(1)–O distances range between 2.463(5) Å for Ag(1)–O(3) and 2.654(5) Å for Ag(1)–O(4). It is found quite often that nitrate ions coordinating the central atom as a bidentate ligand show one short and one significantly longer and weaker bond to the metal atom. The two Ag(1)-carbonyl oxygen bonds to the two coordinating ligand molecules are in the same range: Ag(1)–O(2) 2.516(5) Å and Ag(1)–O(1) 2.623 (5) Å. The same is true for the silver–N bonds Ag(1)–N(2) 2.333(5) Å and Ag(1)–N(3) 2.421(5) Å. If the nitrate ion with its small bite angle of 49.7° is regarded as only one coordinating position, the polyhedron around Ag(1) can be best described as a distorted square-pyramidal coordination with the nitrate ion in apical position. Owing to the bite angles (69.17° for O(2)–Ag(1)–N(3) and 70.36° for O(1)–Ag(1)–N(2)) of the two ligand molecules acting as bidentate ligands too, the basis of the pyramid is a rectangle instead of a square.

The silver site Ag(2) shows a completely different coordination sphere. This silver atom has three bidentate nitrate ions with Ag–O distances gradually ranging between 2.447(5) Å for Ag(2)–O(6) and 2.702(6) Å for Ag(2)–O(7). The coordination sphere is completed by a contact between Ag(2) and C(7), carbon atom belonging to a phenyl group. The distance of 2.690(6) Å is rather short when compared with the sum of the van der Waals radii of 3.42 Å. This contact between the π-electrons of a neutral sp^2^-hybridized carbon atom in an aromatic ring system and an Ag^+^ ion is also found in other compounds [41] although similar Ag–C contacts usually are slightly shorter (approx. 2.5 Å). But it should be taken in account that these values occur when there are no other short bonds between the Ag^+^ ion and polar ligands. In the present case, the silver atom has already three nitrate ions nearby and it seems to be plausible that the Ag–C contact is favoured by the packing of the complex units in the crystal structure. The three nitrate ligands form a distorted triangular environment for the central atom and the phenyl ring has sufficient space above the central atom to provide a carbon atom as the fourth ligand, thus completing a distorted tetrahedral coordination sphere. The distances between Ag(2) to the neighbouring atoms of C(7) (Ag(2)–C(6): 3.179 Å and Ag(2)–C(8): 3.257 Å) are much longer and cannot be considered as bonding interactions although they are still slightly smaller than the sum of the van der Waals radii of silver and carbon.

**Figure 2.**
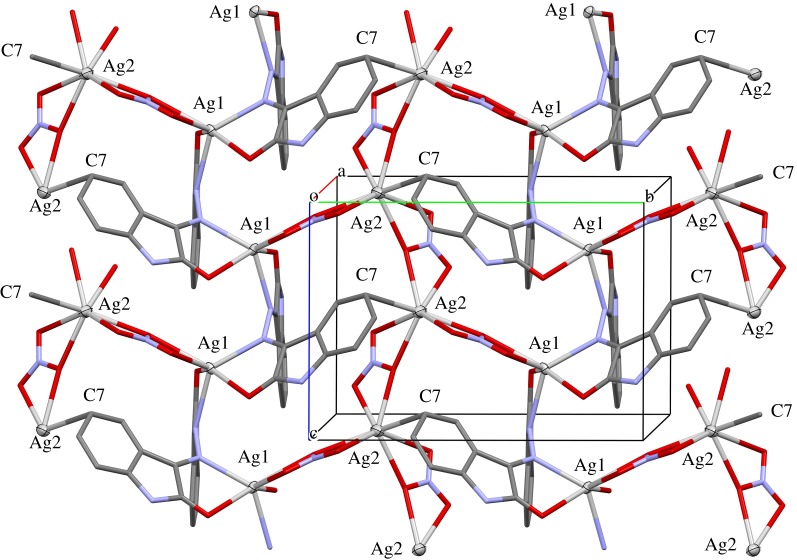
The two-dimensional infinite coordination polymer in the structure of **1** extends perpendicular to the *a*-axis. The numbering of the silver atoms and the atom C(7), which forms a weak bond to Ag(2), is added for a better understanding. All hydrogen atoms are omitted from this figure for better visualization.

The crystal structure contains two-dimensional infinite networks extending perpendicular to the *a*-axis of the unit cell. Figure 2 shows one layer of the network in a view along [100]. The nets are built up by both types of silver atoms as knots and both, the ligand molecule and the two nitrate ions, act as bridging links. As described above, the carbonyl oxygen atoms and the azine nitrogen atoms connect two Ag(1) atoms, additionally one phenyl ring forms a bridge to an Ag(2) site. One oxygen of the nitrate group around N(6) forms a kind of Ag–O backbone by building bridges of the type –O(6)–Ag(2)–O(6)–Ag(2)–O(6)–. The atom O(4) of the nitrate group around N(5) connects each Ag(2) atom with an Ag(1) site. Additionally, to the bridging oxygens each O atom in the nitrate ion coordinates to one silver central atom, as shown in figure 2.

**Figure 3.**
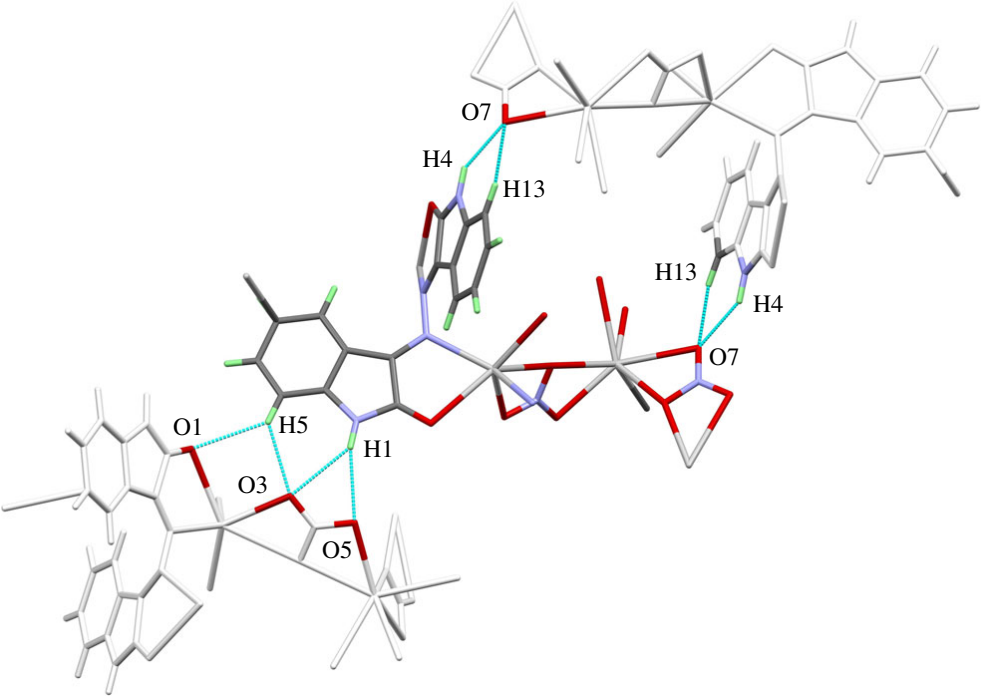
Scheme of the hydrogen bonds connecting various layers of the coordination polymer.

**Figure 4.**
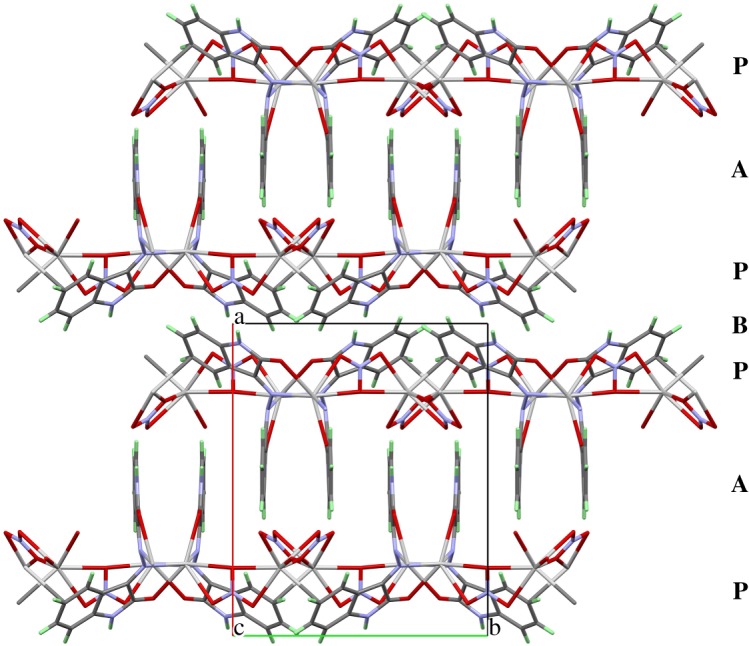
Packing scheme of the complex **1** in a view along the *c*-axis. **P** marks the position of the two-dimensional infinite, polymeric network shown in figure 2. **A** and **B** assign the areas in which the networks interact with another.

Besides the strong interactions of the ligands with the silver central atoms in the two-dimensional infinite networks, there are additional interactions between the complex units. Figure 3 shows that the amine-protons (H(1) and H(4)) as well as some protons bonded to carbon atoms of a phenyl ring (H(5) and H(13)) form hydrogen bridges with the oxygen atoms of the nitrate and carbonyl groups. The hydrogen interactions connect the two-dimensional infinite coordination polymers with another. It can be seen from figure 4 that the packing of the complex in the unit cell leads to two different contact zones between the two-dimensional infinite networks. The area **P** marks the side view of the network structure and the contact zones are assigned by **A** and **B**. In zone **A**, the ring systems of the ligand molecules are all aligned in parallel and partially overlapping if viewed along the *b*-axis. This overlap leads to the formation of some weak π–π stacking interactions. The C–C and C–N distances range between 3.206 and 3.321 Å. The shortest N–H⋯O hydrogen bridge (N(4)–H(4) ⋯O(7): 2.08 Å) also occurs in this part of the structure and connects the two network layers. In Zone **B**, the ring systems of the ligand molecules are folded towards the network plane due to their function as bridging ligands between the silver atoms of the two-dimensional framework. Nevertheless, there is also a number of π–π stacking between adjacent rings. The distances between neighbouring ring systems are slightly larger than in zone **A** and range between 3.396 and 3.535 Å. The networks are also held together by weak hydrogen bridge bond ranging between N(1)–H(1) ⋯O(5): 2.37 Å and C(5)–H(5)⋯O(1): 2.52 Å.

#### 3.2.2. Crystal structure of [Ag_2_L′_2_(ClO_4_)_2_]*_n_*; **2**

**Figure 5.**
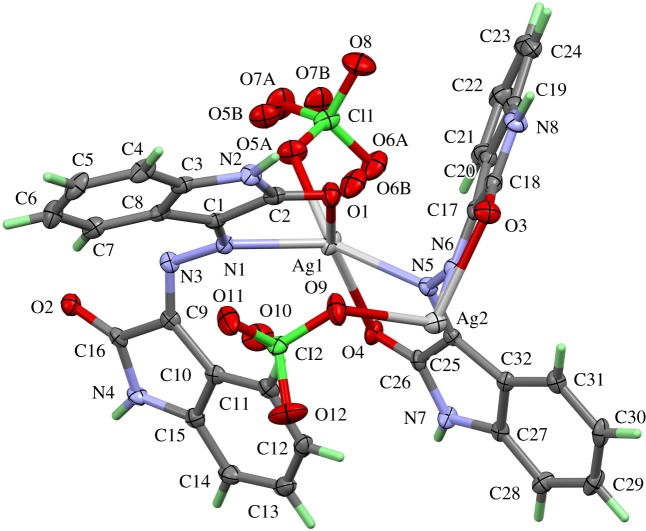
Numbering scheme and atomic displacement ellipsoids drawn at 50% probability level for the asymmetric unit of complex **2**. The atomic numbers of the hydrogen atoms have been omitted for clarity.

The **[Ag_2_L′_2_(ClO_4_)_2_]*_n_*** complex (**2**) of silver with the ligand **L′** crystallizes with one ligand molecule per AgClO_4_ in the triclinic space group *P*-1 and *Z *= 2. Figure 5 shows the content of the asymmetric unit, atom numbering and the displacement ellipsoids. As the structure contains two independent silver sites in each asymmetric unit, the complex has the formula [Ag_2_L′_2_(ClO_4_)_2_]*_n_*. Both silver sites showed similar coordination spheres built up by two chelating ligand molecules and one perchlorate unit. The ligands are coordinated to the central atom via the carbonyl oxygen atom and the nitrogen atom of the azine group was bonded to the five-membered ring system. These four atoms form a distorted rectangular environment for the silver central atoms. The coordination polyhedron is completed by an oxygen atom of the perchlorate group in the apical position of a distorted rectangular-pyramidal environment. The apical oxygen in the coordination sphere around Ag(1) deviates more strongly from its ideal position than the respective site for the polyhedron around Ag(2) because the perchlorate anion around Cl(1) shows an orientation disorder, while the perchlorate group around Cl(2) is perfectly ordered. In the case of the disordered ClO_4_^−^ group, the sites O(5A) and O(6B) coordinate Ag(1) in their respective domains. The silver–nitrogen distances are the shortest contacts for both silver sites. They range between 2.332 Å (Ag(1)–N(5)) and 2.370 Å (Ag(2)–N(3)).

**Figure 6.**
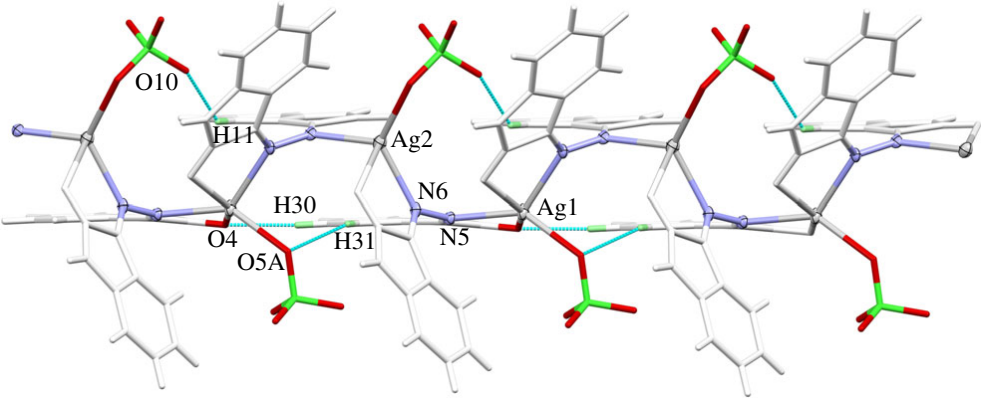
One-dimensional infinite coordination polymer in the crystal structure of complex **2**. The wavy-line shaped Ag–N backbone of the polymeric unit and the positions of the ClO_4_ anions are emphasized. Hydrogen bonds within the coordination polymer are shown as light-blue lines.

The Ag(1) and Ag(2) atoms are connected to another via the N–N– bridges of the azine groups. Figure 6 shows the wavy-line backbone ⋯N–N–Ag–N–N–Ag–N–N⋯ of the one-dimensional infinite coordination polymer in the structure of this complex. The perchlorate anions as well as the organic ring systems of the ligand molecules are arranged in an alternating way along the backbone of silver and nitrogen atoms. One half of the ring systems are almost coplanar—in figure 6, those rings point to the background of the drawing. The other half of the rings on the opposite side of the Ag–N backbone, pointing to the viewer, adopt a fishbone-like arrangement. One of the oxygen atom perchlorate groups (O(10)) forms a weak hydrogen bridge bond with one of the protons (H(11)) of the benzene ring in the coplanar ring part of the coordination polymer.

**Figure 7.**
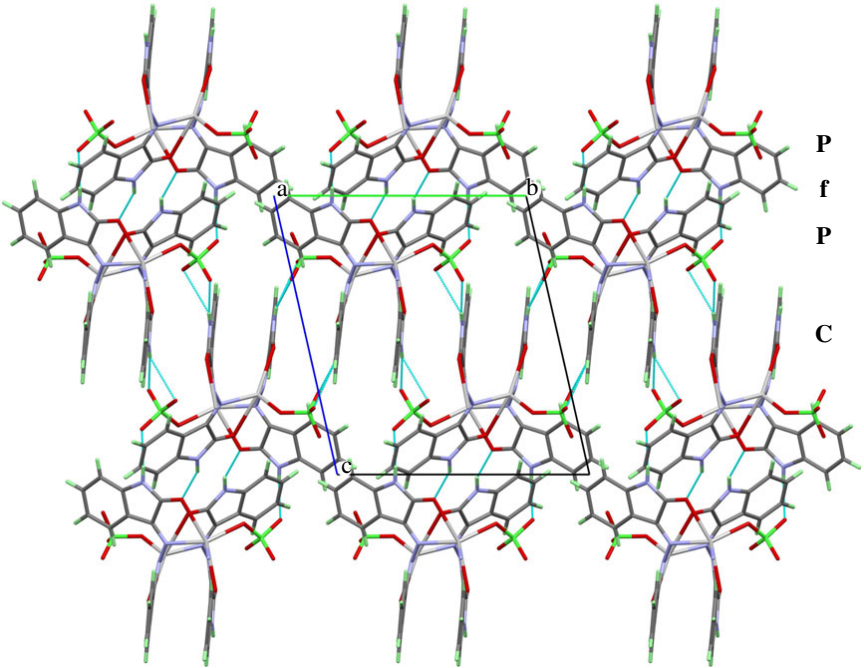
Packing scheme of the perchlorate complex, viewed along the *a*-axis. Hydrogen bond interactions are shown as light-blue lines. **P** marks the position of the coordination polymers, **f** and **c** mark the regions where the fishbone-like ligand parts, respectively, the coplanar ligands interact.

At first glance, the packing scheme of **2** seems to be very similar to that of **1**, but there are significant differences. While the latter contains two-dimensionally infinite sheets of complex units, the former has one-dimensional infinite strings of complexes that are packed side by side along the *a*-axis and interact only weakly by van der Waals contacts. In the region **f** shown in figure 7, the coplanar parts of the ligand molecules interact mainly via π-stacking interactions and a few weak hydrogen bridges between the perchlorate anions and protons of the benzene rings. The fishbone-like ligand parts allow a much shorter distance between the coordination polymer backbones. The contacts between the strings are dominated by strong hydrogen bridge bonds between the carbonyl oxygen atoms and amine-protons of adjacent molecules.

#### 3.2.3. Crystal structure of [Ag(Isatin-3-hydrazone)NO_3_]; **3**

**Figure 8.**
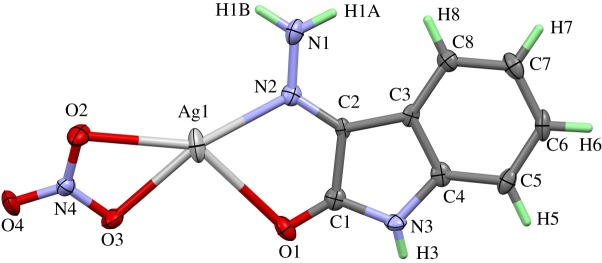
Numbering scheme and atomic displacement ellipsoids drawn at 50% probability level of **[Ag(Isatin-3-hydrazone)NO_3_]** complex; **3**.

This complex crystallizes in the monoclinic space group *P*2_1_/*c* with four formula units per cell. The content of the asymmetric unit including numbering scheme and displacement ellipsoids are shown in figure 8.

The silver atom in this monomeric complex is coordinated by one isatin-3-hydrazone molecule and one nitrate ion, both acting as bidentate ligands. The coordination sphere is best described as a distorted, square planar environment. Owing to the small bite angles of the nitrate (50.77°) and the hydrazone molecule (69.73°), the distortion of the coordination sphere is rather strong. The distances from the central atom to its ligand atoms vary strongly from 2.263 Å for Ag(1)–N(2) to 2.667 Å for Ag(1)–O(1). Further details regarding the X-ray structure of this complex are available in electronic supplementary material, figures S2–S4.

#### 3.2.4. Crystal structure of the ligand **L**′

**Figure 9.**
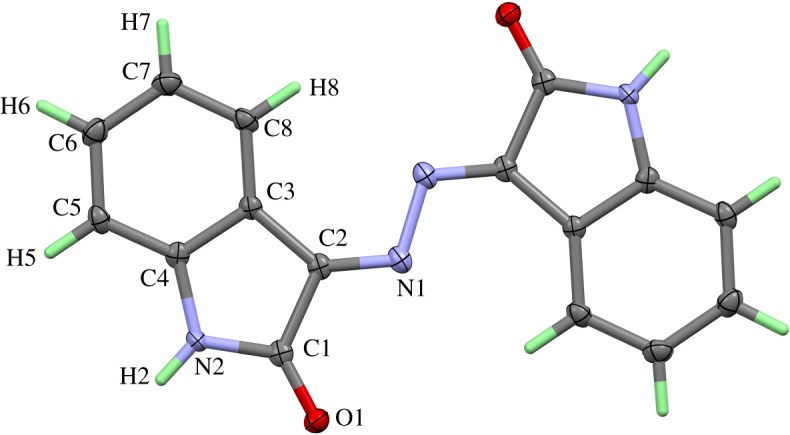
Numbering scheme and atomic displacement ellipsoids drawn at 50% probability level of the ligand molecule **L′**. This molecule is located on a centre of symmetry, thus the asymmetric unit only contains the labelled atomic sites.

The ligand **L′** crystallizes in the high symmetric, trigonal space group *R*-3 with nine formula units per cell. The molecule is situated on a centre of symmetry, thus the asymmetric unit contains only a half molecular unit of **L′** (figure 9). The centre of symmetry is located in the centre of the azine group, N–N bond. The packing structures of the **L′** molecules are presented in detail in the electronic supplementary material. A few years ago, Liu *et al*. described the crystal structure of this compound [42] with the same symmetry using room temperature data and 0.17 H_2_O per formula unit of the ligand molecule. In the current case, no water molecules in the voids of the structure around the threefold axes were observed. Details regarding this point and the packing of **L′** are available in electronic supplementary material, figure S5.

### 3.3. Hirshfeld analysis of molecular packing

**Figure 10.**
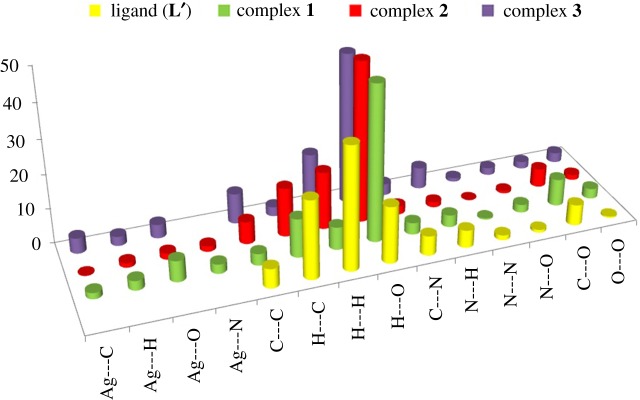
The percentages of all possible intermolecular contacts in the crystal of the studied compounds**.**

The Hirshfeld surface analyses shown in electronic supplementary material, figures S6–S14 are used to give descriptive and quantitative clarifications about the intermolecular interactions in the crystal [28,43–45]. Quantitative summary of all possible interactions is presented in figure 10. It is clear that the common and most important intermolecular interaction in the crystal structure of the studied compounds is the O⋯H hydrogen bond. These interactions contributed by 16.5, 43.6, 45.1 and 46.3% from the overall fingerprint plots of the ligand (**L′**), **1**, **2** and **3**, respectively. Complex **3** showed some weak intermolecular Ag⋯C interactions (Ag–C; 2.803–3.291 Å), while **1** has one strong Ag2–C7 (2.690 Å) interaction. In complex **2**, no Ag⋯C interactions were observed. Based on the shape index (SI) and curvedness maps, the free ligand has no characteristics of any significant π–π stacking interactions. By contrast, the silver complexes showed some π–π stacking interactions, where the SI maps have the complementary red/blue triangles (see the black ellipse in electronic supplementary material, figure S6) characteristics for the π–π stacking interactions.

### 3.4. Atom in molecules and natural bond orbital analyses

**Figure 11.**
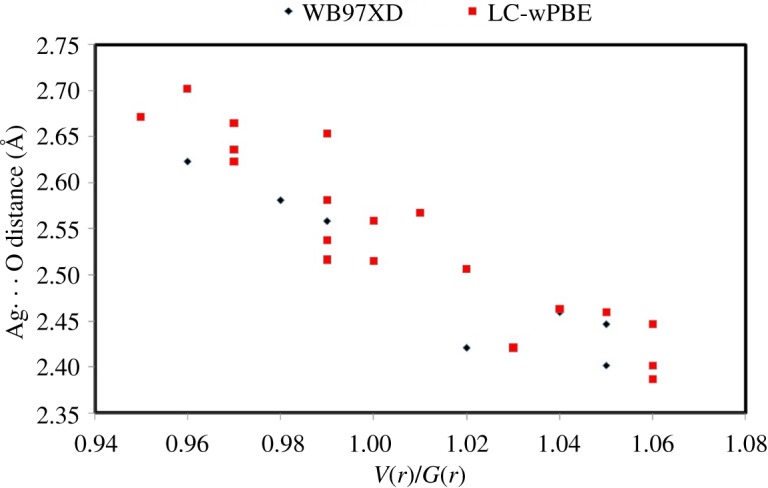
Variations of the *V*(*r*)/*G*(*r*) ratio with Ag⋯O distances in the studied complexes.

In this section, the AIM topological parameters (electronic supplementary material, table S2) are employed to inspect the nature and relative strength of the Ag–N, Ag–O and Ag–C interactions. Using the three DFT methods, the presence of bond critical point (BCP) at the bond baths (Ag–O, Ag–N or Ag–C) between the interacting atoms is an indicator on the presence of significant interaction among them where the higher electron density *ρ*(*r*) [46] at the BCP is an indicator of strong interaction. Complexes **1** and **2** have similar patterns of Ag–N interactions with one weaker and one stronger Ag–N bond per **L′**. This revealed very well from the higher interaction energies (*E*_int_ = *V*(*r*)/2) [47] of the shorter bonds than the longer ones. The Ag–O interactions showed wide variations of *ρ*(*r*) ranging from 0.0192 to 0.0405 atomic units (au) for Ag2-O17 (2.702 Å) and Ag2-O16 (2.387 Å). Generally, the *ρ*(*r*) values decay significantly with increasing the Ag–O distances. Interestingly, the presence of BCP at the Ag2–C7 bond bath in **1** with *ρ*(*r*) = 0.0222 au revealed the presence of significant Ag–C interaction. On the other hand, the ratio of |*V*(*r*)|/*G*(*r*) and the total energy density *H*(*r*) values is a good indicator for the bond covalent characters [48]. The negative *H*(*r*) values and |*V*(*r*)|/*G*(*r*) ratio greater than 1 indicated some covalent character for all the Ag–N and Ag–C interactions. It is clear that the Ag–O interactions have *H*(*r*) values ranging from −0.0037 to 0.0013 au and |*V*(*r*)|/*G*(*r*) ratios close to 1 (0.95–1.06). As shown in figure 11, there is an increase in the bond covalent character with a decrease in the Ag–O distances.

**Figure 12.**
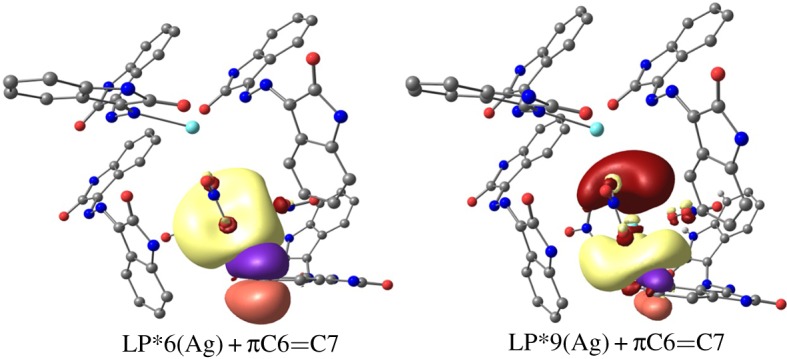
The natural orbitals included the Ag–C7 interaction in **1**. Yellow and brown for the Ag-orbitals, while orange and violet for the ligand donor atoms NBO.

Moreover, NBO analysis is performed in order to show the most important Ag-orbitals involved in the Ag–N, Ag–O and Ag–C interactions. The energies due to the donor (NBO*_i_*)–acceptor (NBO*_j_*) interactions are given in detail in electronic supplementary material, tables S3 and S4. It is clear that the dispersion effect is important in describing the Ag–N, Ag–O and Ag–C interactions where both the WB97XD and the LC-wPBE showed that not only the LP*6Ag, mainly s-orbital character is the important anti-bonding orbital included in these interactions, but also the LP*7Ag, LP*8Ag and LP*9Ag played important roles which are totally neglected in the B3LYP calculations; the latter have no dispersion correction. Pictorial presentation of these natural orbitals for complex **1** as an example is shown in electronic supplementary material, figure S15. The Ag–C interaction occurs mainly between the filled π-type filled orbital of the C6=C7 and both of the LP*6 and LP*9 anti-bonding orbitals of Ag2 (figure 12). On the other hand, the intramolecular charge transfer from donor NBOs (ligand/anion) to the acceptor one (Ag(I)) affects the occupancy and the energies of the NBOs (electronic supplementary material, table S5). The silver(I) anti-bonding orbitals are increased in occupancy up to 0.202 e and significantly stabilized. Most of this effect occurred for the LP*(6)Ag. On the other hand, the energy and occupancy of the filled ligand/anion natural orbitals are affected little due to these interactions. Generally, the occupancy of the ligand orbitals is lowered and either stabilized or slightly destabilized.

## 4. Conclusion

Via self-assembly, three silver(I) complexes were synthesized by direct mixing of isatin oxamohydrazide (**L**) and silver(I) salts in the water/methanol solution at room temperature. The isolated crystals were identified using SC-XRD measurements to be [Ag_2_L′(NO_3_)_2_]*_n_*, [Ag_2_L′_2_(ClO_4_)_2_]*_n_* and [Ag(Isatin-3-hydrazone)NO_3_], where **L′** is the azine-type ligand of isatin. Only silver(I) catalysed the conversion of isatin oxamohydrazide (**L**) to **L′** via the formation of isatin-3-hydrazone. Testing different metal ions ended with the recovery of the isatin oxamohydrazide (**L**) either at room temperature or by heating under reflux for 24 h. Also, the acidic medium catalysed the conversion of the isatin oxamohydrazide to the corresponding azine one (**L′**). [Ag_2_L′(NO_3_)_2_]*_n_* and [Ag_2_L′_2_(ClO_4_)_2_]*_n_* are two- and one-dimensional coordination polymers, while the [Ag(Isatin-3-hydrazone)NO_3_] consists of disconnected complex molecules. Hirshfeld analysis was used to quantify the intermolecular interactions in the crystals of the synthesized compounds, while AIM and NBO methods were used to describe the coordination interactions between Ag(I) and the ligand donor atoms.

## Supplementary Material

CIF and CheckCIF.rar

## Supplementary Material

Supplementary Information

## Data accessibility

The datasets supporting this article have been uploaded as part of the electronic supplementary material. It includes spectroscopic measurements (FTIR and NMR spectra) of the studied compounds, details regarding the single crystal structure of **3** and **L′** as well as the most important DFT results. Also, it includes the CIFs and CHECKCIFs. Electronic supplementary material is available from the CCDC upon request, quoting the deposition numbers CCDC-1548202 (**1**), CCDC-1548204 (**2**), CCDC-1548200 (**3**) and CCDC-1548201 (**L′**). Measurements at room temperature are also deposited with CCDC numbers of 1548203 (**1**), 1548205 (**2**) and 1548199 (**3**). These data can be obtained free of charge at www.ccdc.cam.ac.uk/conts/retrieving.html or fax: (internat.) +44-1223/336-033; e-mail: deposit@ccdc.cam.ac.uk.
