# Supplementary material for: Unexpected formation of polymeric silver(I) complexes of azine-type ligand via self-assembly of Ag-salts with isatin oxamohydrazide
Source: R Soc Open Sci. 2018 Jul 25;5(7):180434. doi: 10.1098/rsos.180434 (PMC6083671; doi:10.1098/rsos.180434)
Supplement: Supplementary Information [file rsos180434supp2.docx]

**Unexpected Formation of Polymeric Silver(I) complexes of azine-type ligand via self assembly of Ag-salts with isatin oxamohydrazide.**

**Saied M. Soliman^1,2^, Jorg H. Albering^3^ and Assem Barakat^4^**

^1^Department of Chemistry, Faculty of Science, Alexandria University, PO Box 426

Ibrahimia, 21321 Alexandria, Egypt

^2^Department of Chemistry, Rabigh College of Science and Art, King Abdulaziz

University, Jeddah, Saudi Arabia

^3^Graz University of Technology, Mandellstrasse 11/III, 8010 Graz, Austria Q1

^4^Department of Chemistry, College of Science, King Saud University, PO Box 2455,

Riyadh 11451, Saudi Arabia

*Corresponding Author: Tel:+203-5917883, Fax: +‏203-5932488, E-mail: [Saied1soliman@yahoo.com](mailto:Saied1soliman@yahoo.com)

**Figure S1 FTIR and ^1^H NMR spectra**

**Ligand (L')-FTIR**

IR spectra were measured as KBr pellets on a Nicolet 6700 FT-IR spectrophotometer.

**
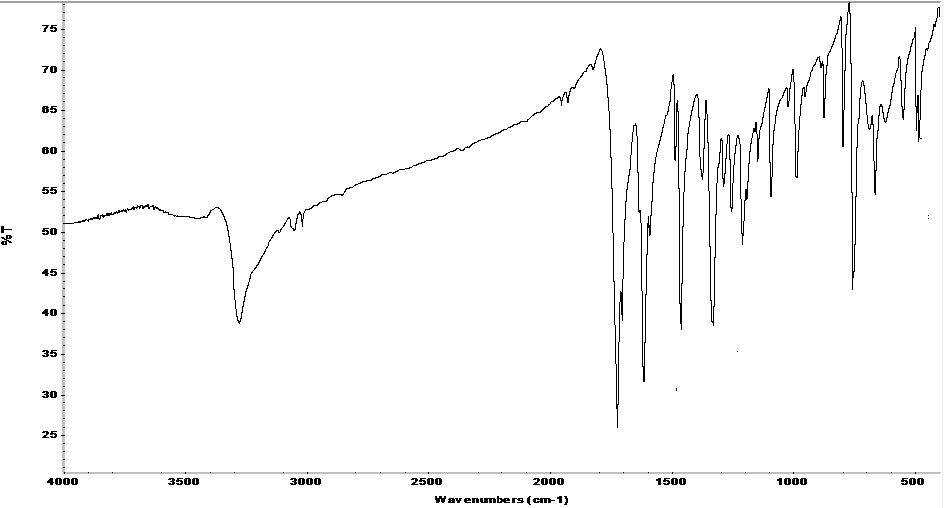
**

**[Ag_2_L'(NO_3_)_2_]_n_-FTIR**

**
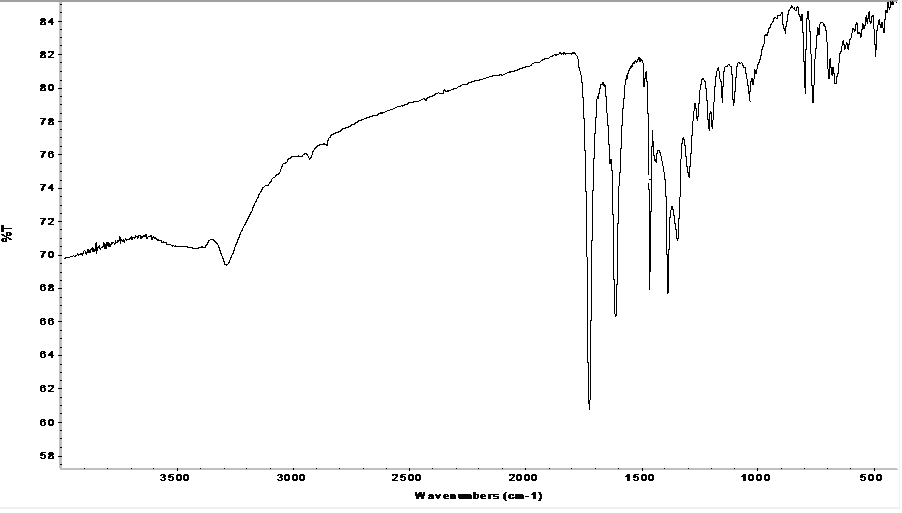
**

**[Ag_2_L'_2_(ClO_4_)_2_]_n_-FTIR**

**
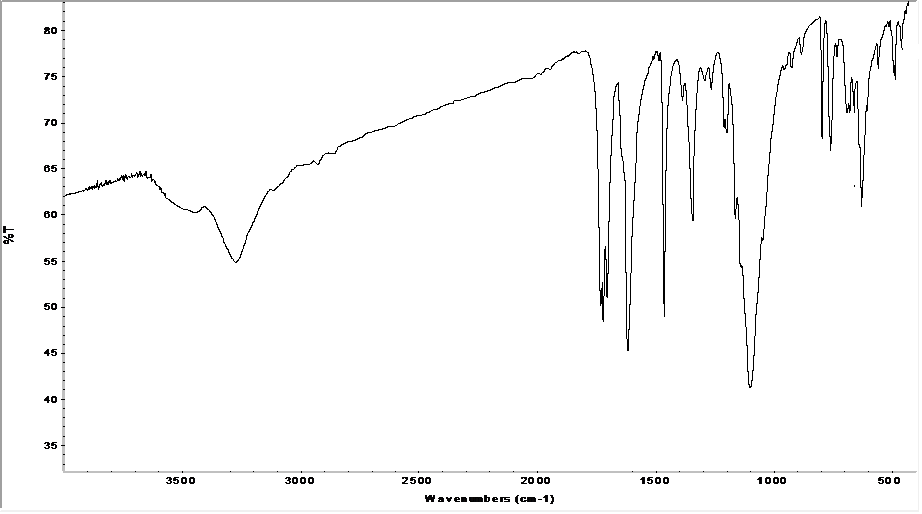
**

**[Ag(Isatin-3-hydrazone)NO_3_] -FTIR**

**
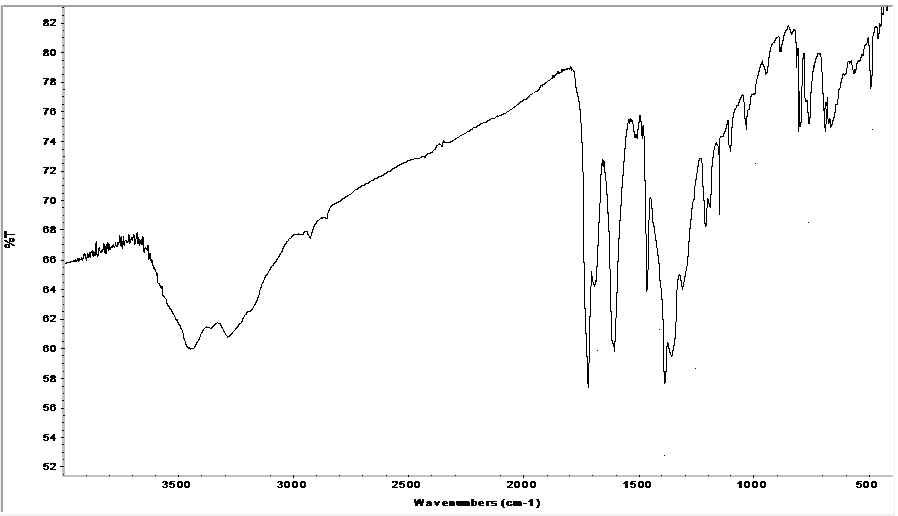
**

**Ligand L'-^1^H NMR**

**
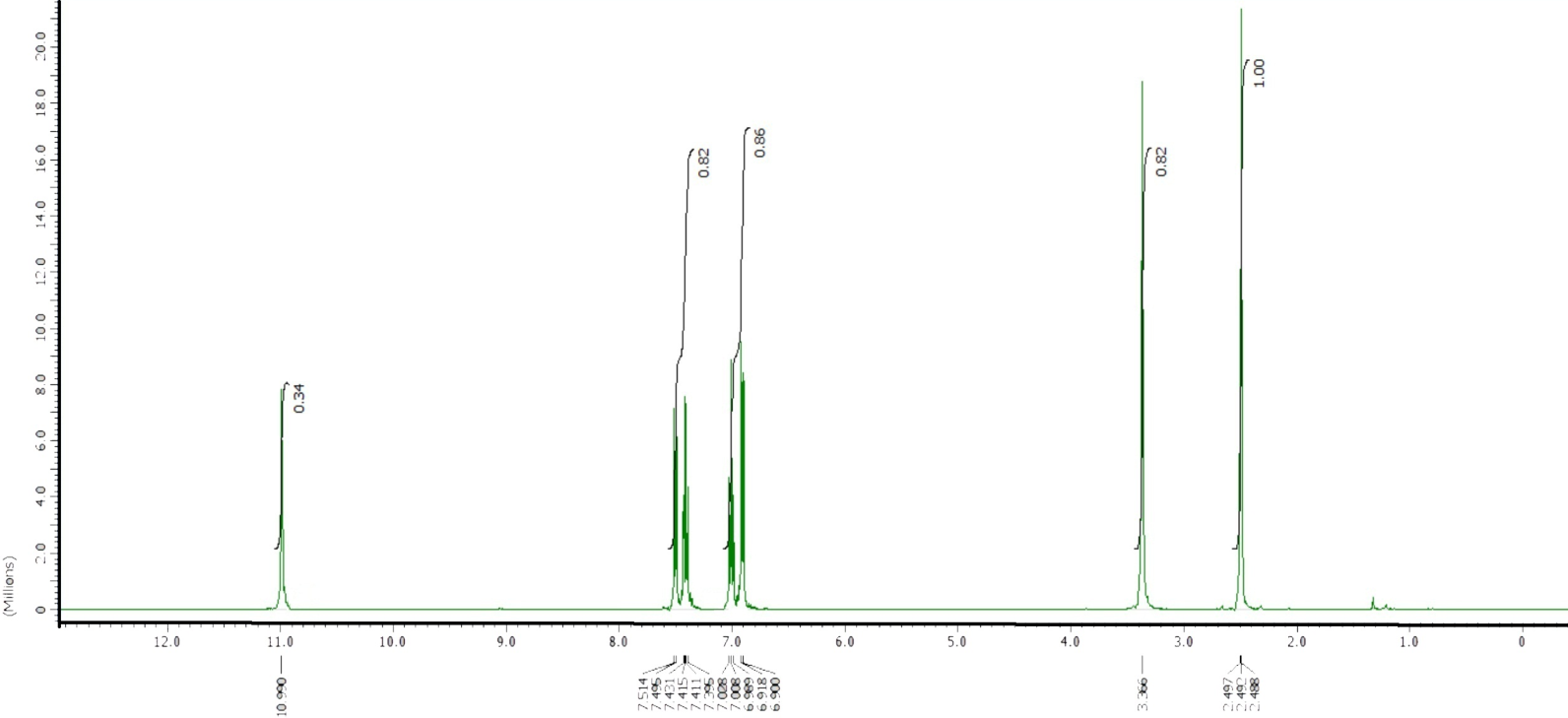
**

**[Ag_2_L'(NO_3_)_2_]_n_-^1^H NMR**

**
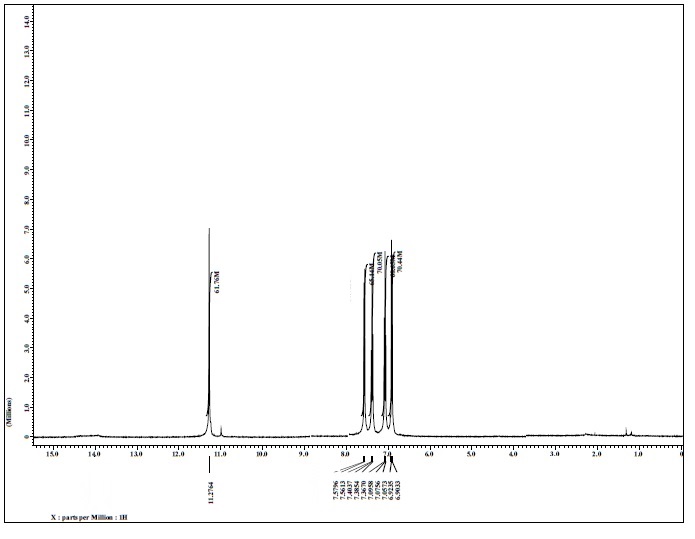
**

**[Ag_2_L'_2_(ClO_4_)_2_]_n_-^1^H NMR**

**
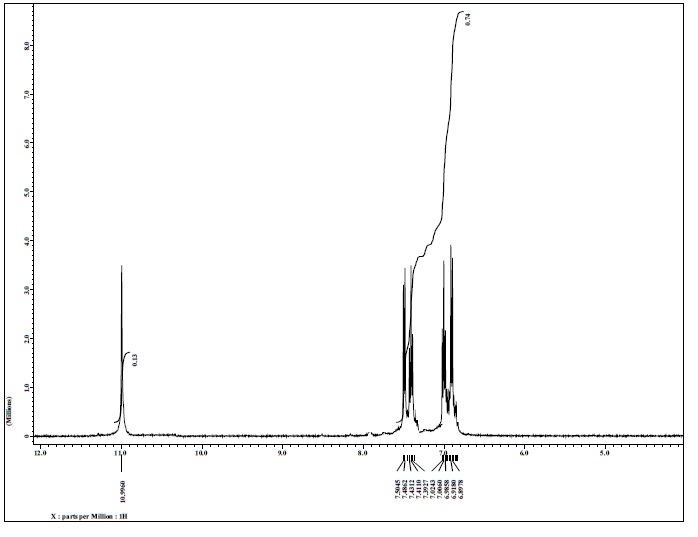
**

**[Ag(Isatin-3-hydrazone)NO_3_] -^1^H NMR**


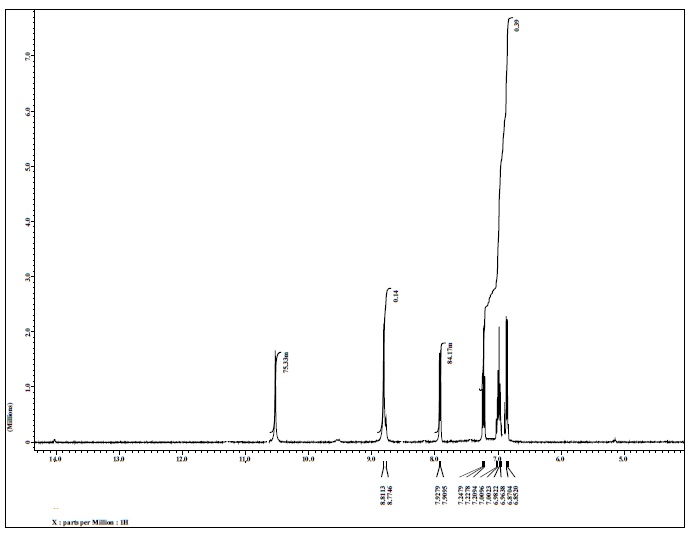


**Isatin oxamohydrazide L-^1^H NMR**

**Detailed description of the molecular packing of 3**

As can be seen from **Figure S1**, the silver atoms have two more neighbouring atoms at rather long distances, augmenting the coordination sphere to a 4+2 stretched octahedral arrangement. The Ag-C and Ag-O distances of Ag(1)-C(6): 2.803 Å resp. Ag(1)-O(4): 2.924 Å surely are too long to be considered as a full bonding interaction, but they might be regarded as “contact” or weak interactions. As compared to the site Ag(2) in the nitrato complex **(1**), (2.690Å), the Ag-C distance is slightly longer in the structures of **3**, but still much shorter than the sum of the van der Waals radii of silver and carbon. The shortest distance between the coplanar nitrate anions occurs between N4-O3: 2.917 Å. The coplanar complexes form stacks along the *a*-axis with alternating slightly longer and shorter distances between the ligand ring systems (**Figure S1)**. The “pair” of complexes with shorter distances is marked by light blue lines representing the shortest occurring distances between two adjacent molecules. The complex molecules in these pairs are oriented head-to-tail. The silver atoms of each complex unit show interactions with the C(6) atom of the neighbouring coplanar ligand. Additionally, the pairs are weakly connected to another by the formation of a C(6)-H(6)…O(4) hydrogen bridge bond. Regarding the distance to adjacent pairs of complexes, it is remarkable, that the distance between the anions is significantly shorter (2.917 Å) than between the π-stacked ligand molecules (centroid distance 3.423 Å; all interatomic distances are longer than this value).


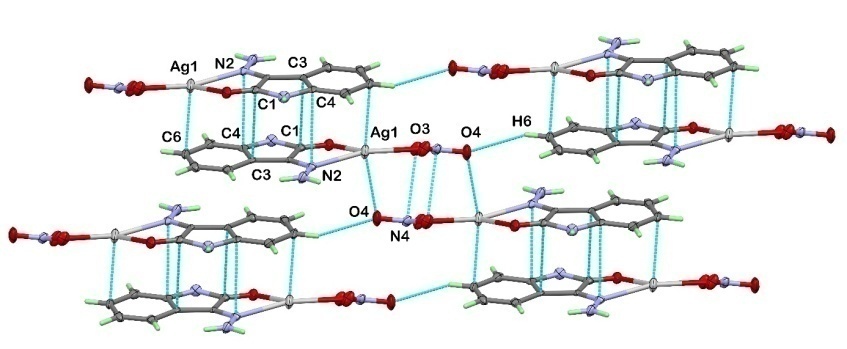


**Figure S2** Interactions between the monomeric **[Ag(Isatin-3-hydrazone)NO_3_]** complex units: Ag1-C6: 2.803 Å, N2-C4: 3.202 Å, C1-C3: 3.301 Å, Ag1-O4: 2.924 Å, N4-O3: 2.917 Å and very weak O4-H6 interactions: 2.69 Å. These double-stacks of complex units show π-stacking interactions with the units above and below. The distance between the centroids (not shown in this figure) of the adjacent five- and six-membered rings is 3.423 Å.

Besides the ligand-silver bonds, the polar contacts between the O and N atoms of the nitrate ions and the π-stacking interactions, there are numerous hydrogen bridge bonds occurring in the crystal structure, connecting the complex units with another. The C(6)-H(6)…O(4) interaction has already been mentioned; the other hydrogen bonds can be seen in **Figure** **S2**. The nitrate ions act as connectors between the complex units by formation of H-bonds to the NH_2_ group as well to the NH group in the five-membered ring. Additionally, some C-H protons of the six-membered ring are also involved in the formation of weak hydrogen bond. The carbonyl oxygen atom O(1) is also involved in building up the hydrogen bond network by the formation of a strong interaction with one of the amine protons H(1B). **Figure S3** shows a projection of the crystal structure along [100]. It can be seen, that the stacks of complex units is connected to another by a complex system of various hydrogen bond interactions, thus forming a 3-dimensional H-bonding network.


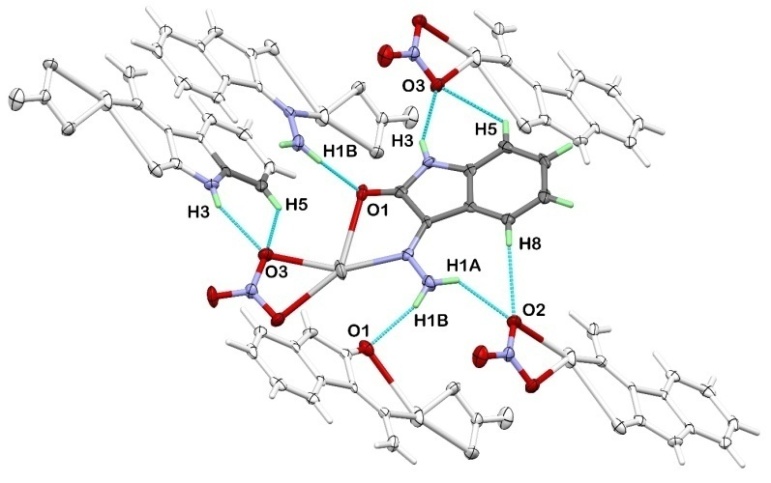


**Figure S3** Hydrogen bonds (light-blue, broken lines) occurring in the structures of **3**.

**
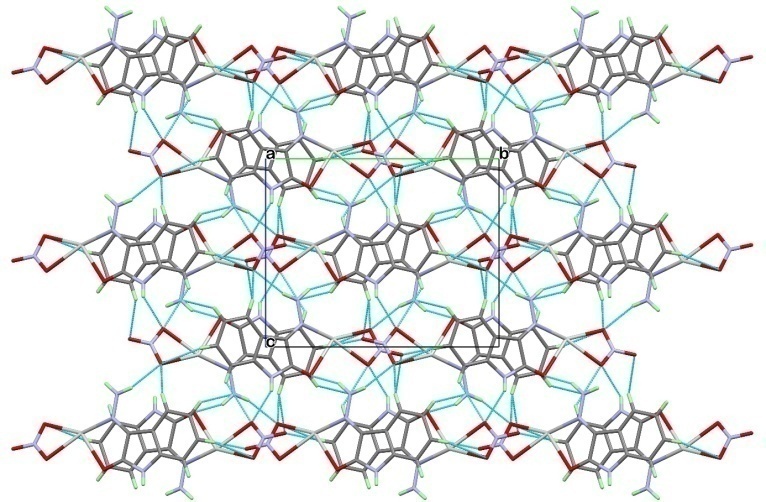
**

**Figure S4** Packing scheme of **[Ag(Isatin-3-hydrazone)NO_3_]:** view along stacks of monomeric complex units extending along the a-axis. Numerous O…H and N…H interactions between the monomers lead to the formation of a complex H-bonding network.

**Detailed description of the molecular packing of L'**

The ring systems in the S-shaped ligand **L'** are coplanar, which means that the π-electrons can be delocalized over the whole molecule. The rings bonded to the azine group are in trans position to another. In the unit cell, the flat molecules are arranged in stacks of coplanar molecular units, which are in contact to another by weak π-stacking interactions. These stacks are connected to another by some hydrogen bridges building-up a loose 3-dimensional network. **Figure S4** contains the projection of the crystal structure along the crystallographic *c*-axis. The hydrogen bridge network is emphasized by the blue lines representing the interactions of the carbonyl O with H-N and H-C groups. The potentially solvent accessible channels in the structure discussed above extend along the threefold axes [001], [1/3, 2/3, 1] and [2/3, 1/3, 1].


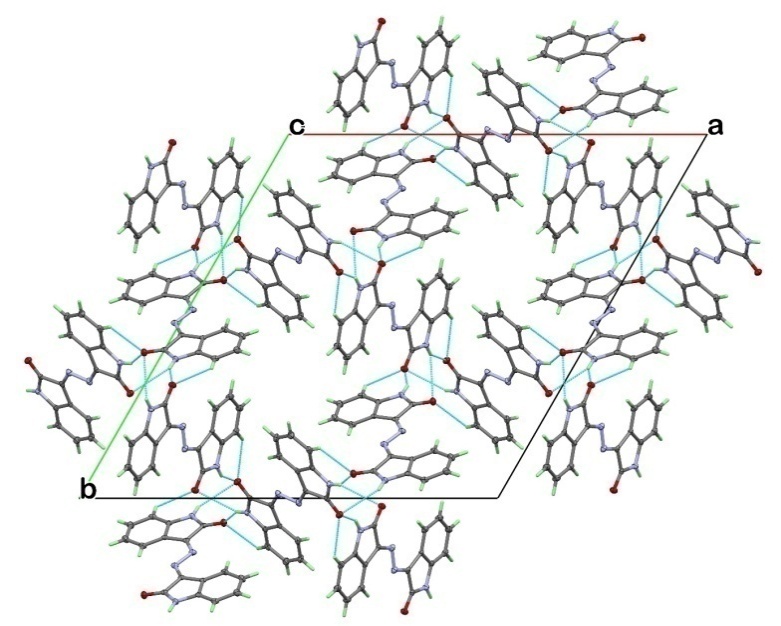


**Figure S5** Projection of the ligand **L'** crystal structure along the trigonal *c*-axis. The light-blue lines represent hydrogen bonding interactions between the stacks of ligand molecules. The empty space around the trigonal axes of the structure contains only insignificant amounts of electron density, indicating that no or only extremely small amounts of solvent molecules are present.

In comparison with the structure reported by Liu et. al in reference [26], the highest electron densities - indicating the hydrogen atoms of the benzene ring - adopted values of 0.7 – 0.82 electrons/Å^3^, while there was only one peak in the electron density map at 1/3, 2/3, 0.42 with an electron density 0.16 e^-^/Å^3^. Even after adding the benzene protons to the structure model, the residual electron densities indicating the bonding electron in the ring systems were much higher than in the voids available for solvent molecules. Thus, we conclude that the presence of water molecules in the channel type voids is optional, possibly depending on the preparation and crystallization method, but not obligatory.

**Figure S6** The Hirshfeld surfaces of the studied compounds, the d_norm_ map range is -0.488 to 2.327.

| 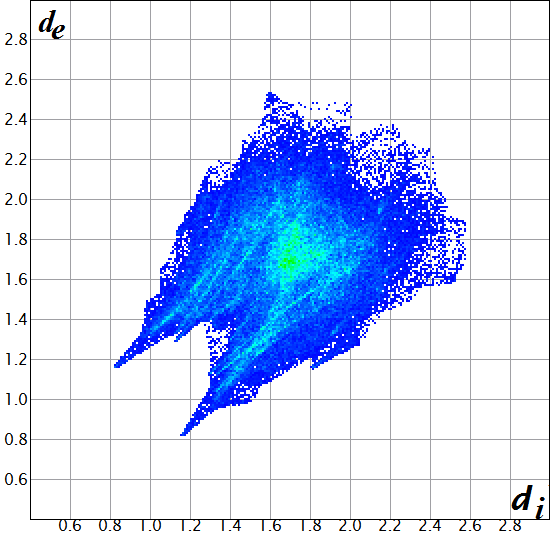 | 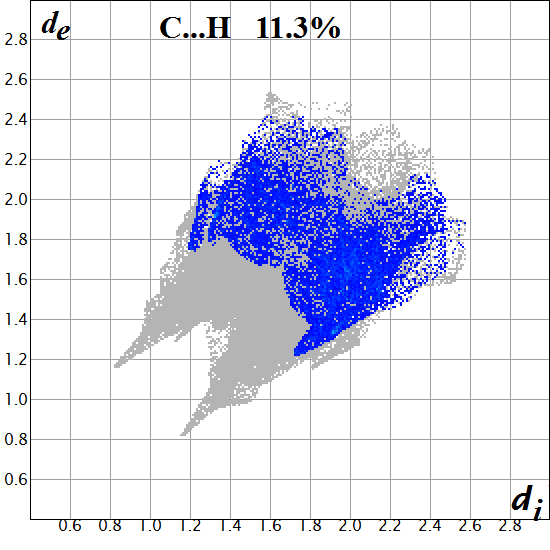 | 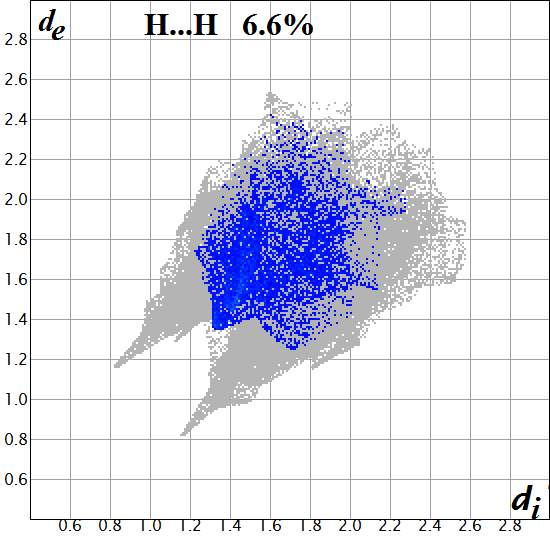 |
| --- | --- | --- |
| 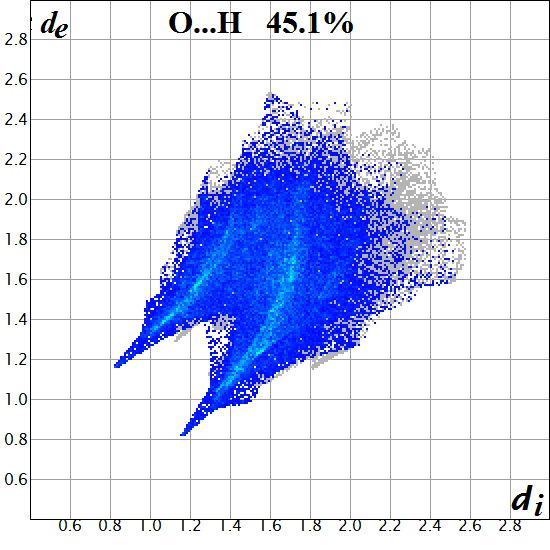 | 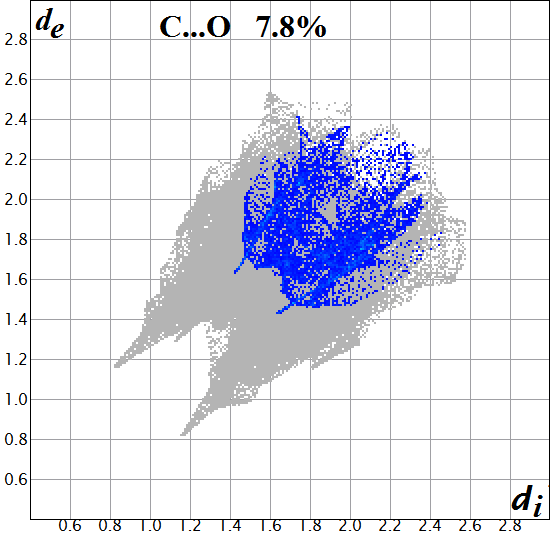 | 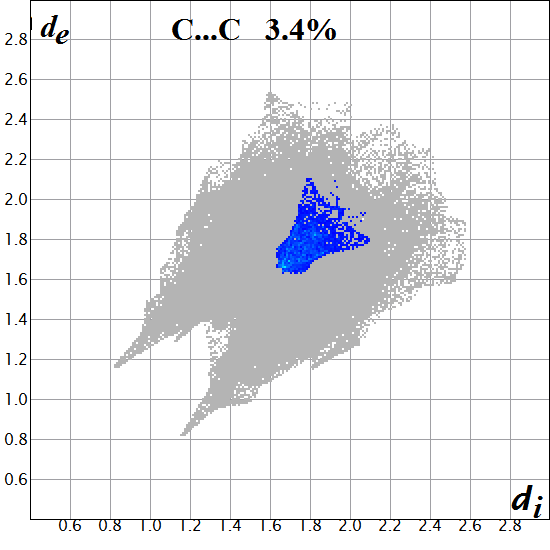 |
| 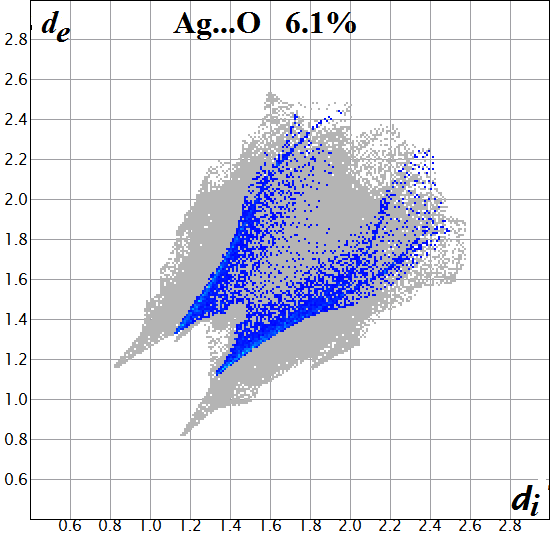 | 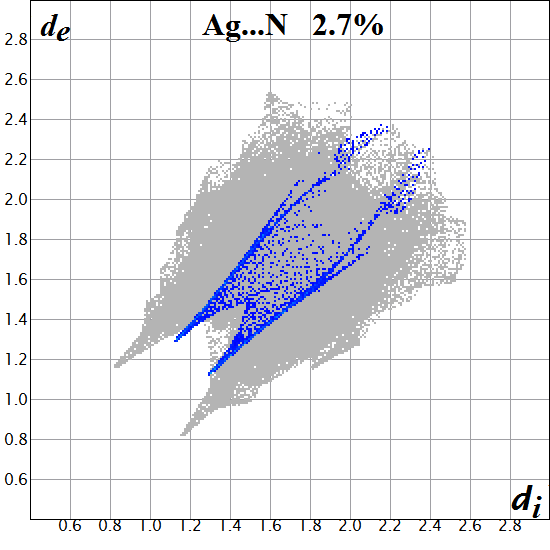 | 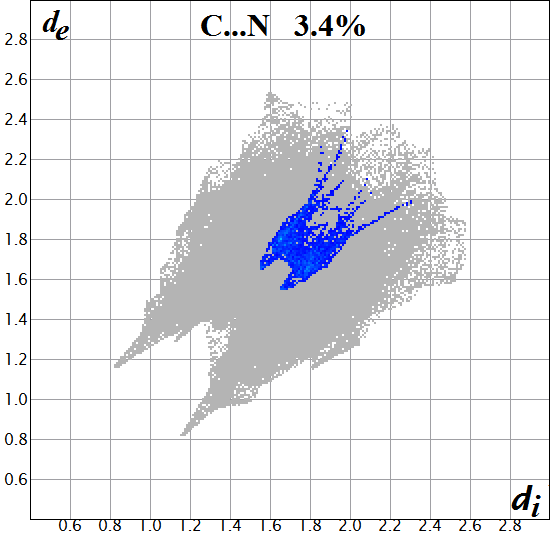 |
| 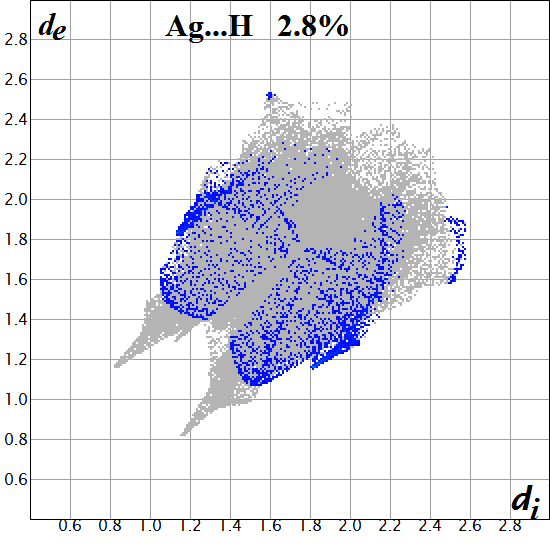 | 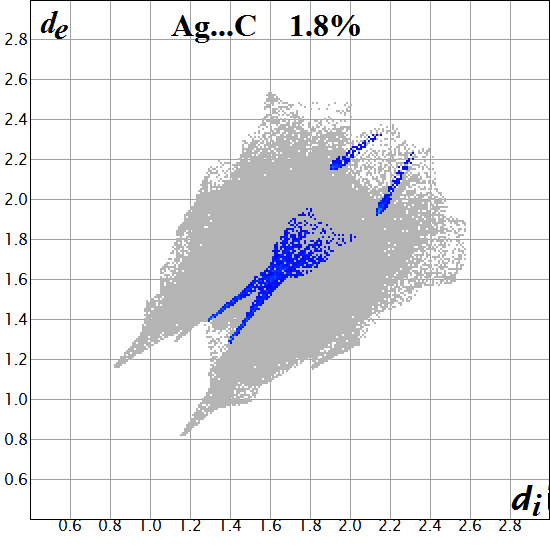 | 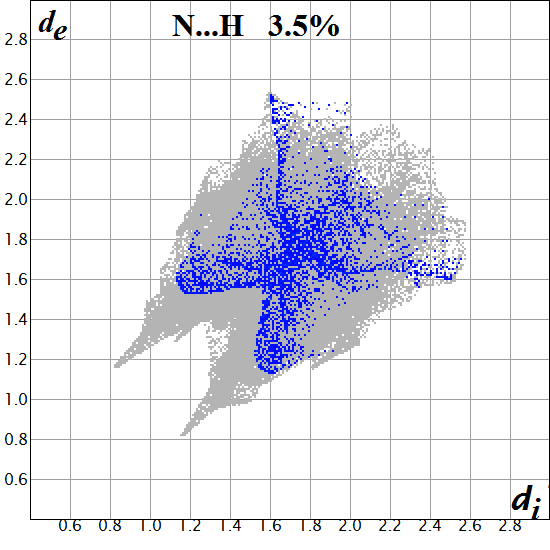 |
| 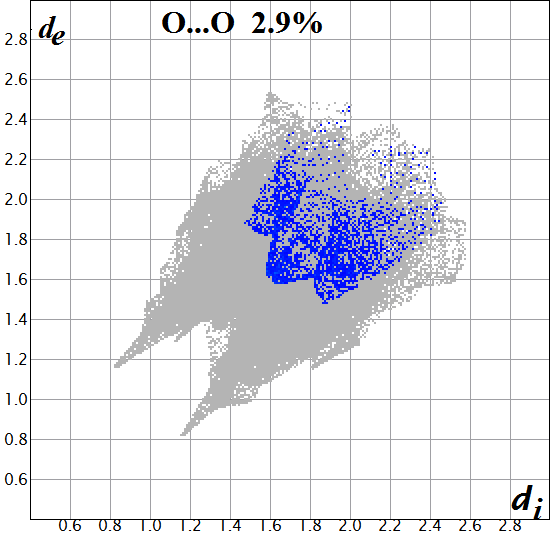 | 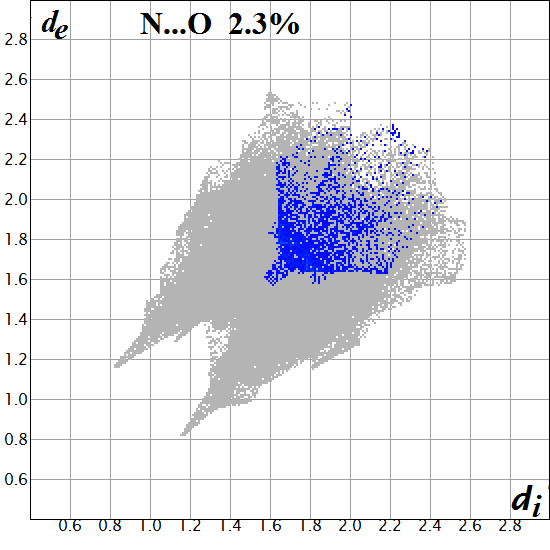 | 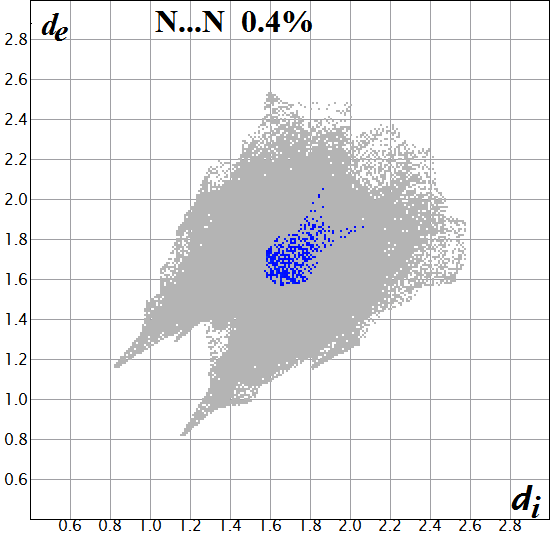 |

**Figure S7** The decomposed fingerprint plots of complex **1**.

| 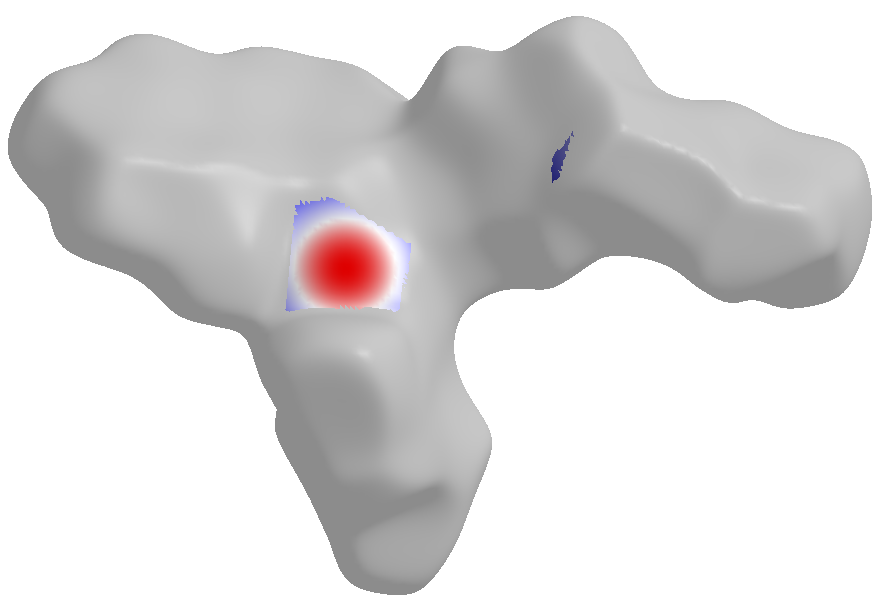 | 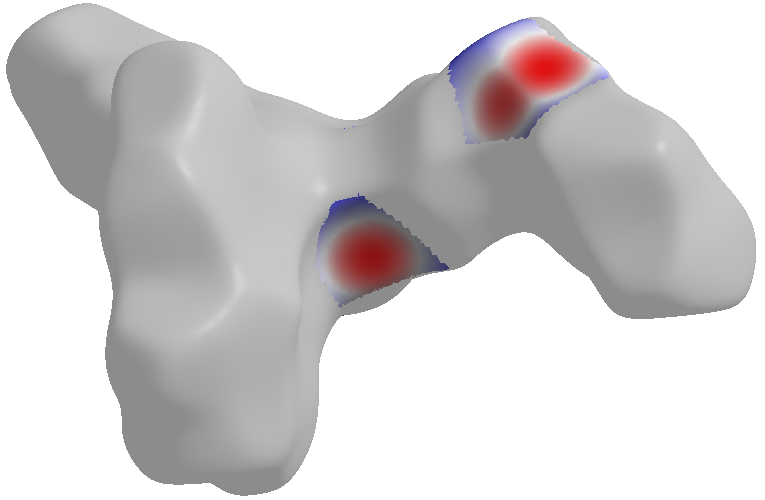 | 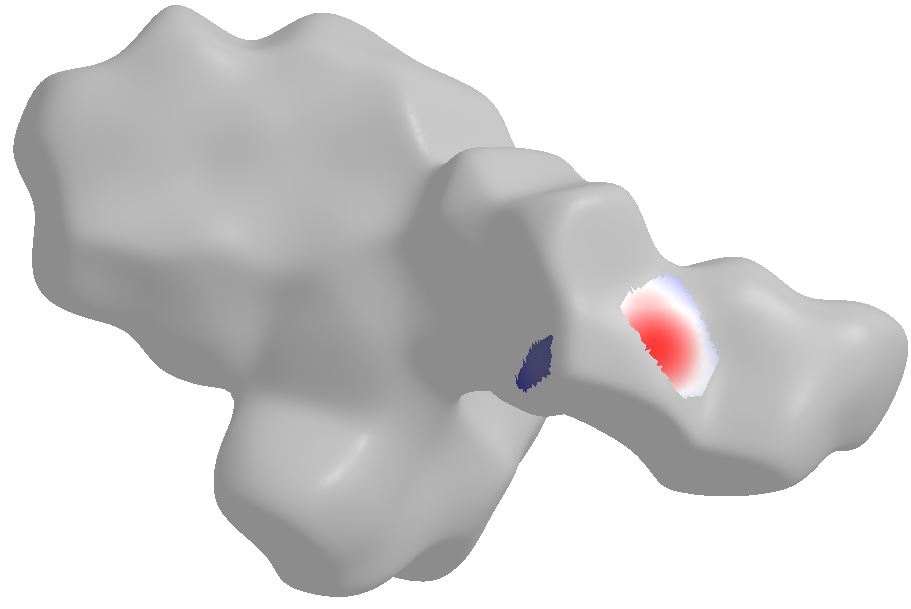 |
| --- | --- | --- |
| **Ag1-N3(2.420ÅÅ)** | **Ag-O(2.460-2.665 Å)** | **Ag2-C7(2.690Å)** |
| 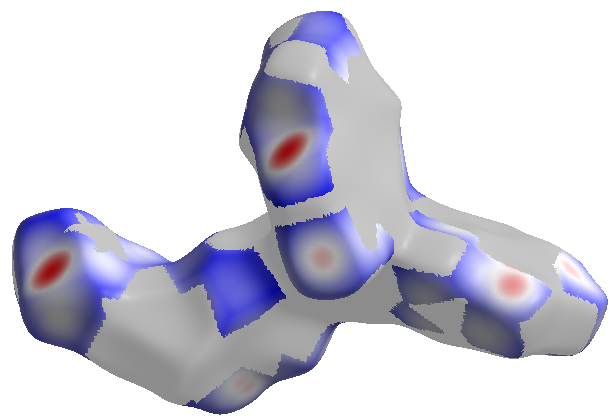 | 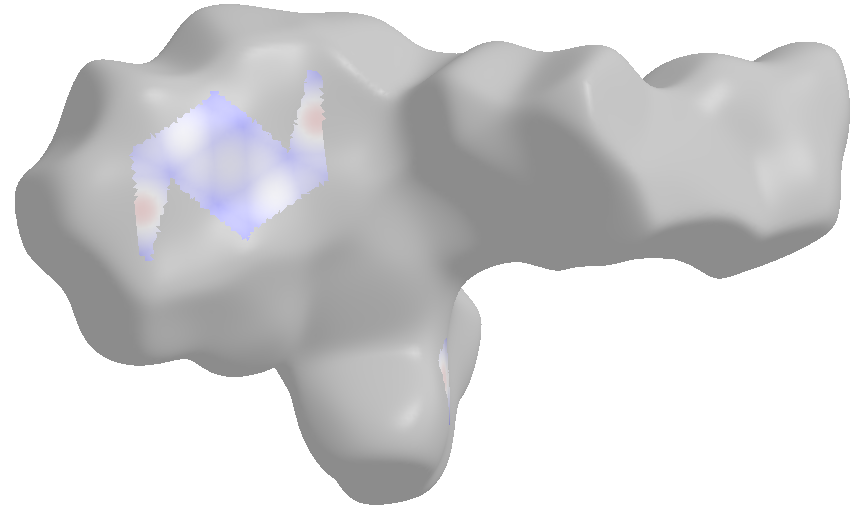 | 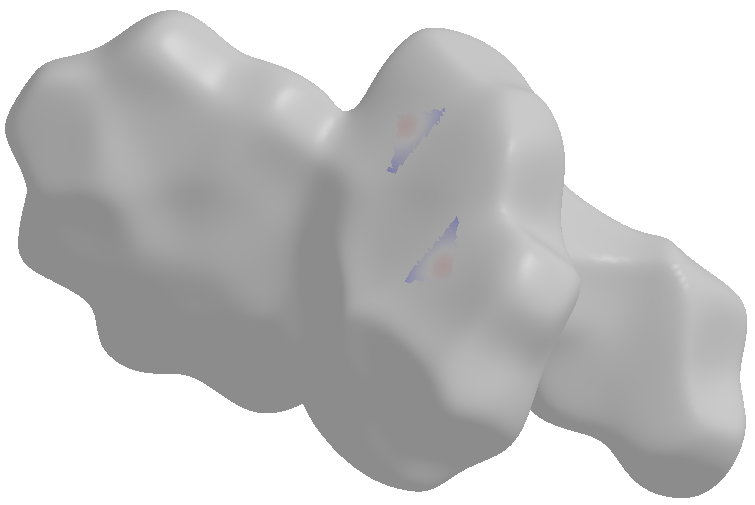 |
| **H4...O7(1.977Å)** | **C1....C6 (3.305Å)** | **C9...C13 (3.321Å)** |

**Figure S8** The decomposed d_norm_ maps of complex **1**.

| 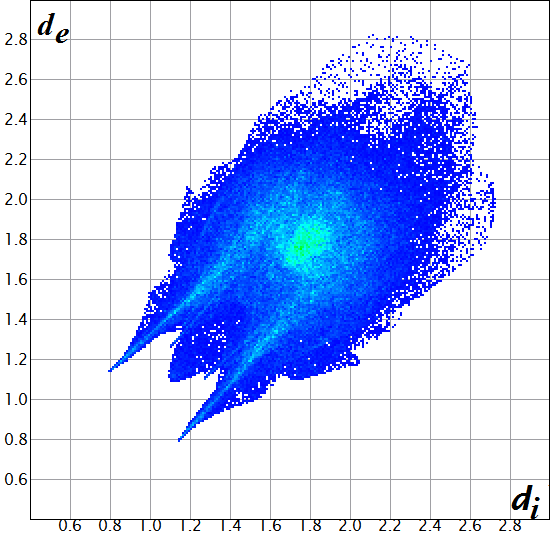 | 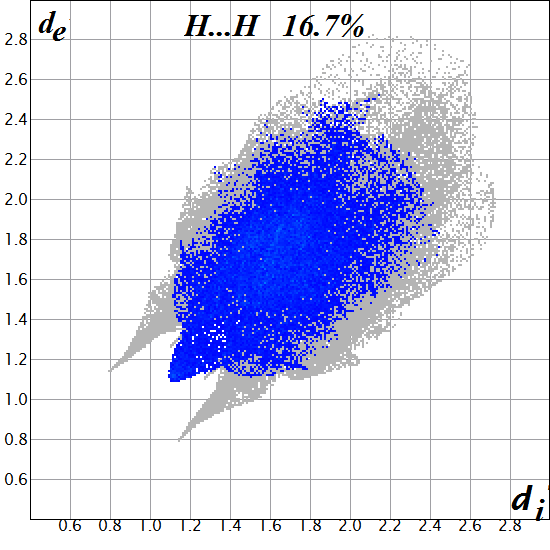 | 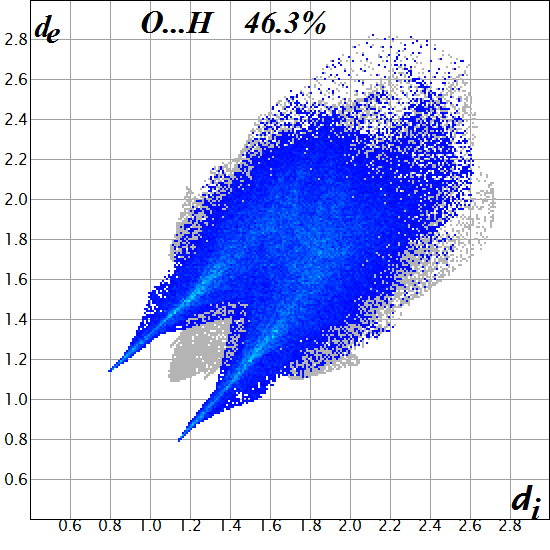 |
| --- | --- | --- |
| 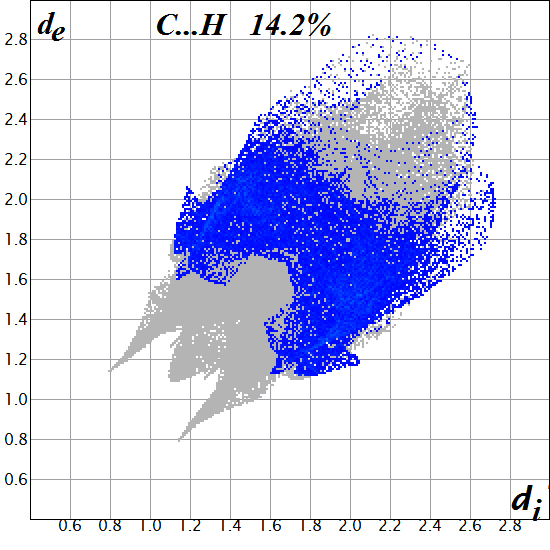 | 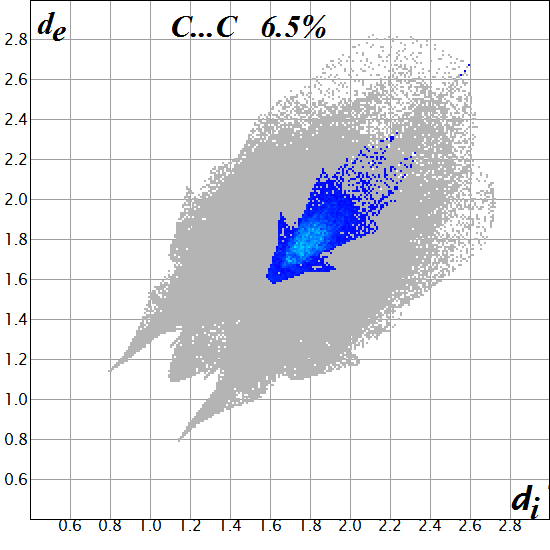 | 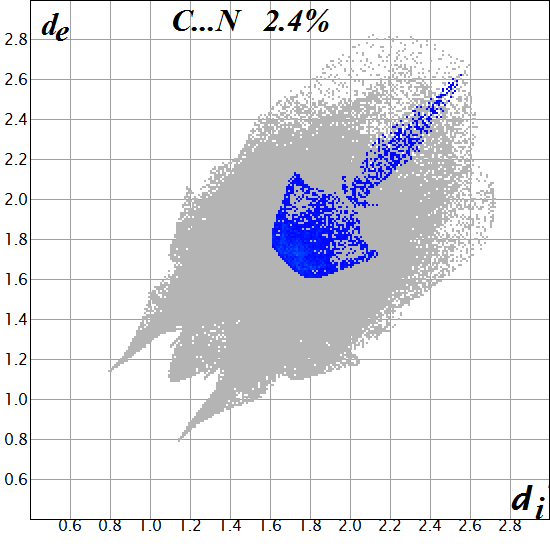 |
| 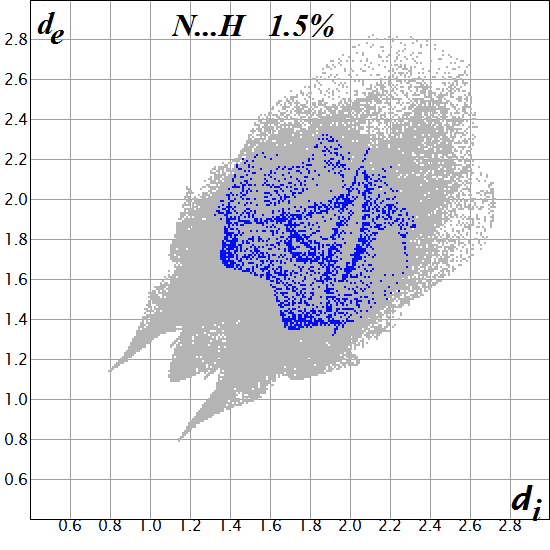 | 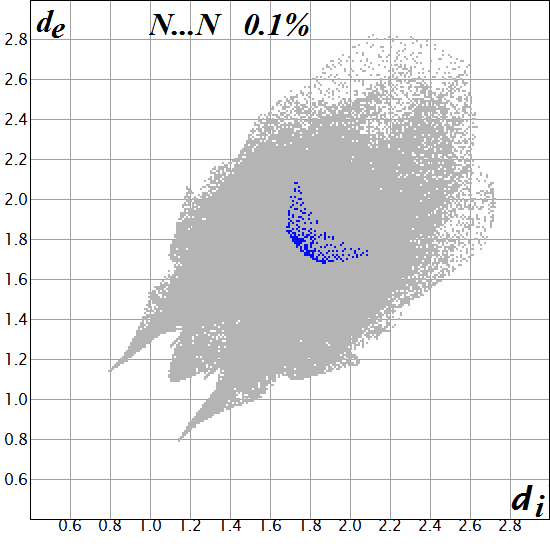 | 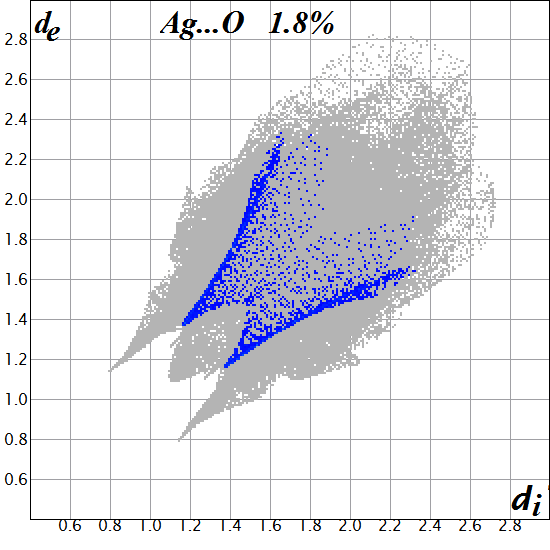 |
| 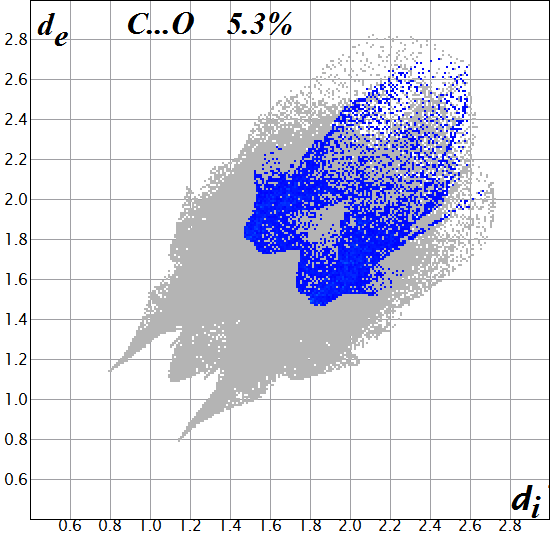 | 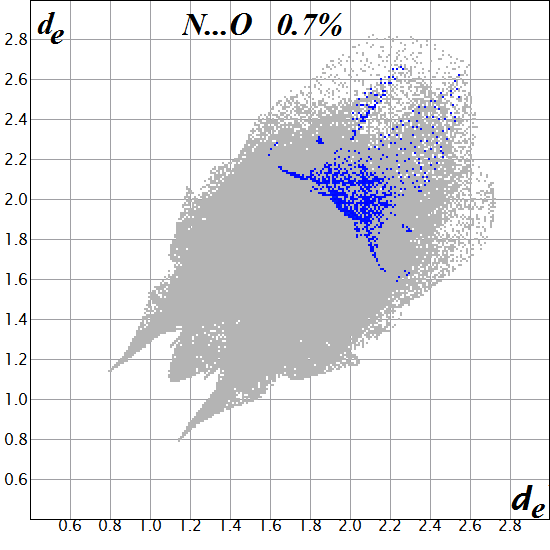 | 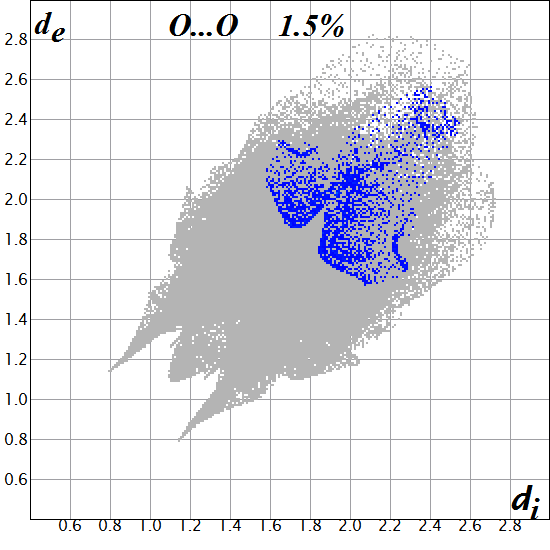 |
| 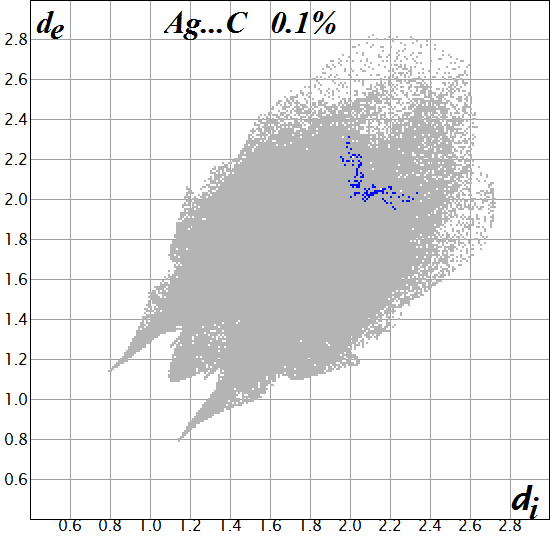 | 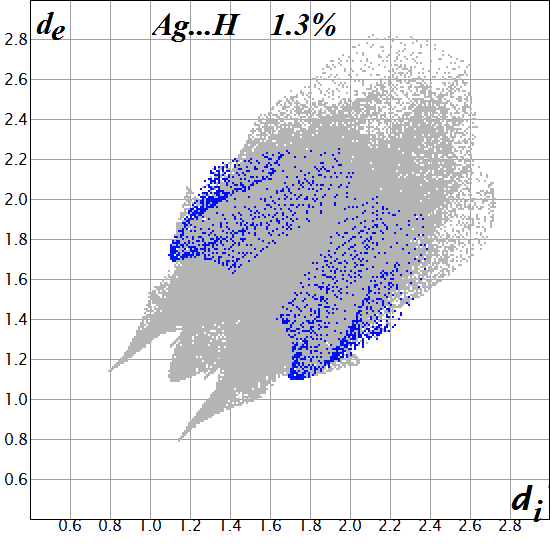 | 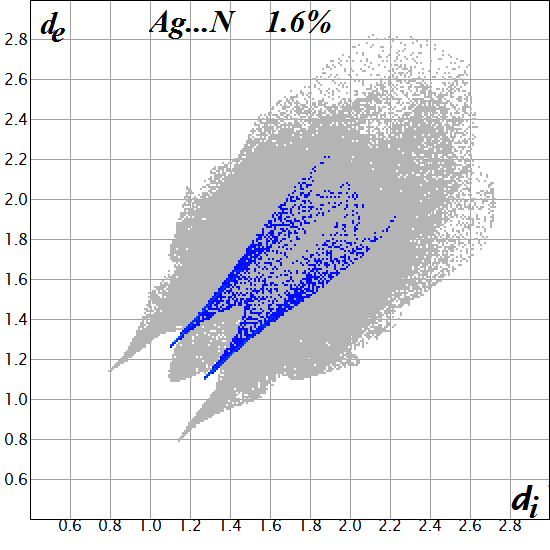 |

**Figure S9** The decomposed fingerprint plots of complex **2**.

| 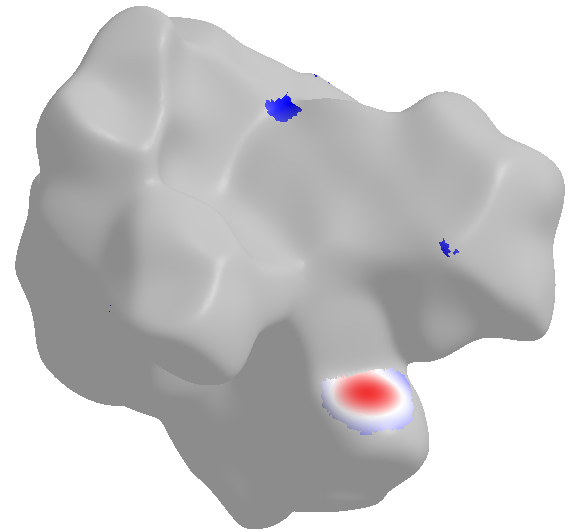 | 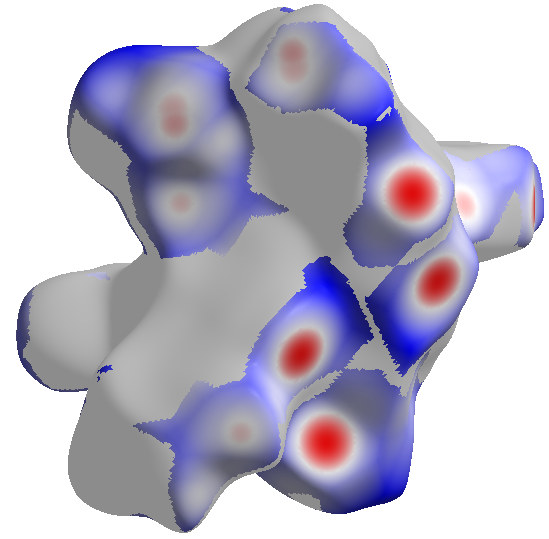 | 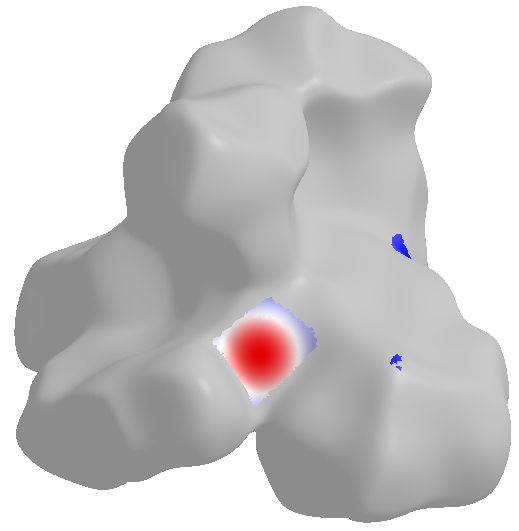 |
| --- | --- | --- |
| **Ag2...O2(2.538 Å)** | **O...H (1.934-1.957Å)** | **Ag2-N3(2.370Å)** |
| 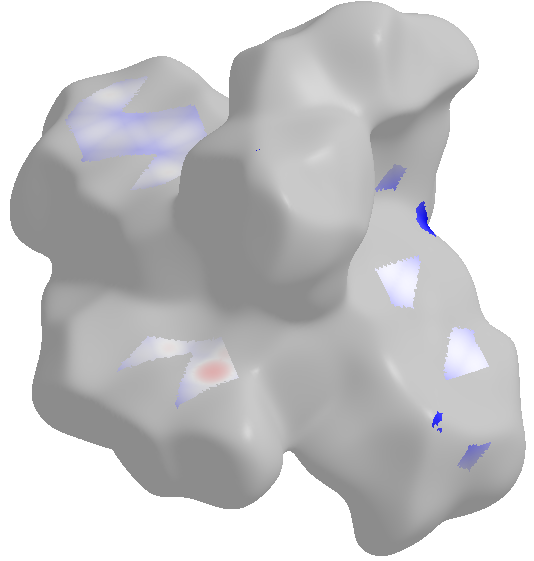 | 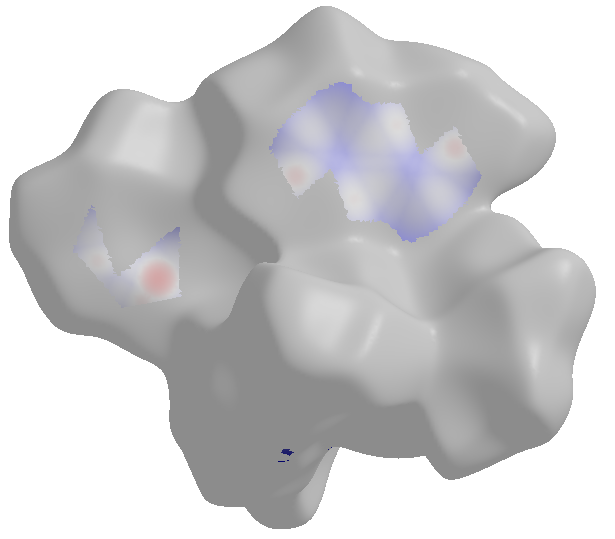 |  |
| **C16...C31(3.200 Å)** | **C...C(3.327A-3.459Å)** |  |

**Figure S10** The decomposed d_norm_ maps of complex **2**.

| 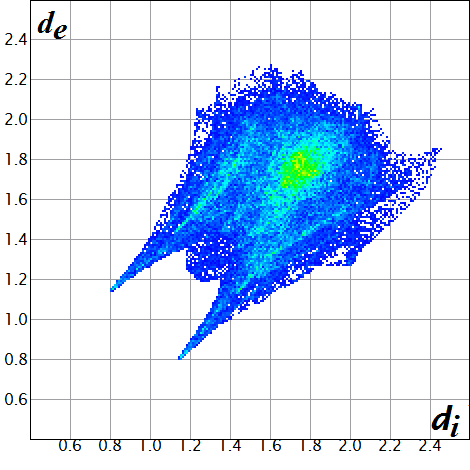 | 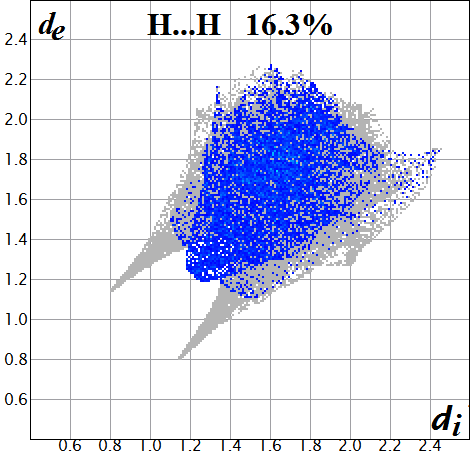 | 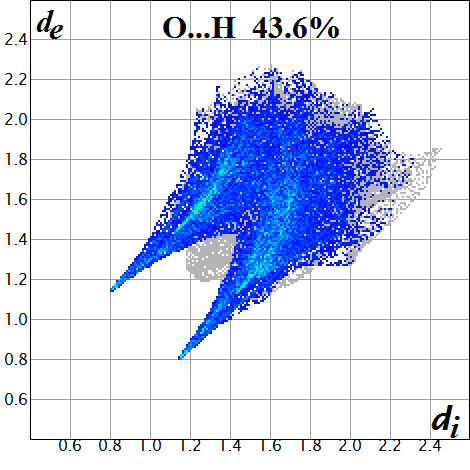 |
| --- | --- | --- |
| 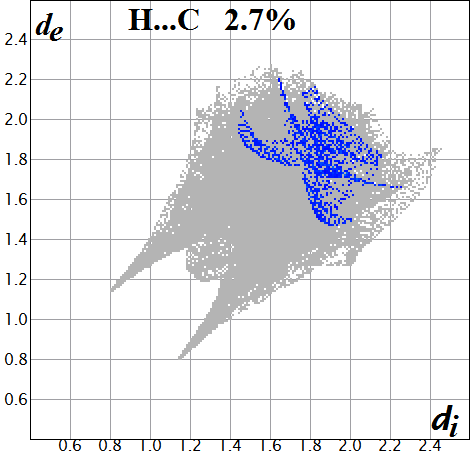 | 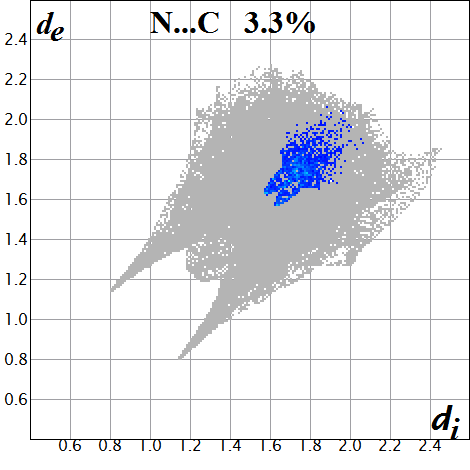 | 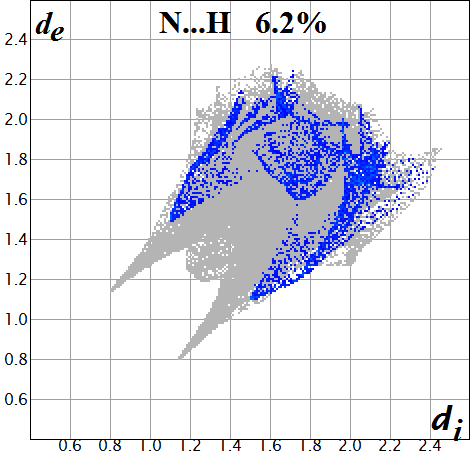 |
| 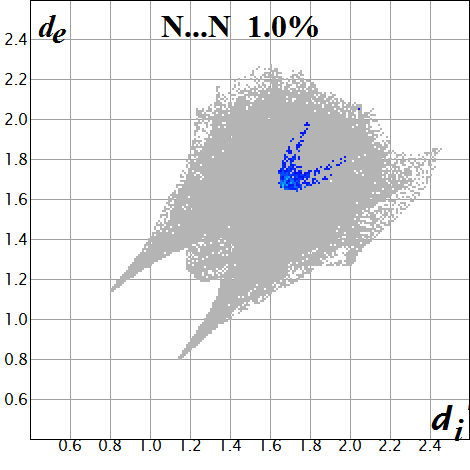 | 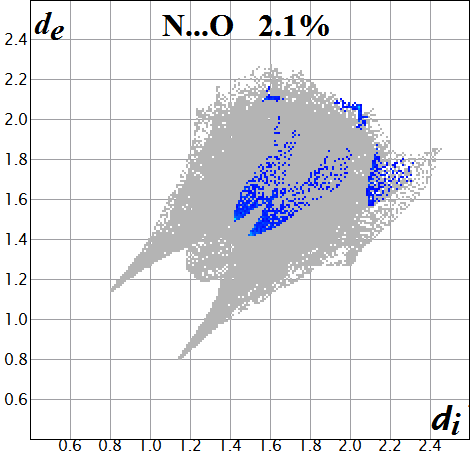 | 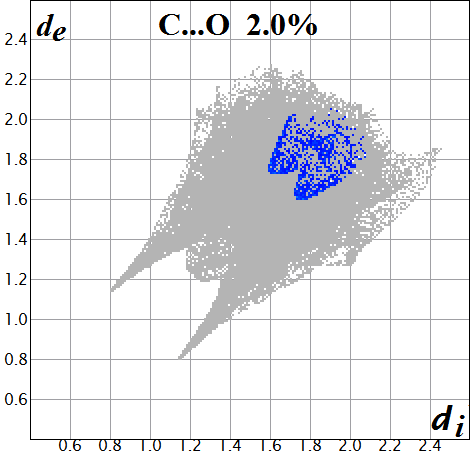 |
| 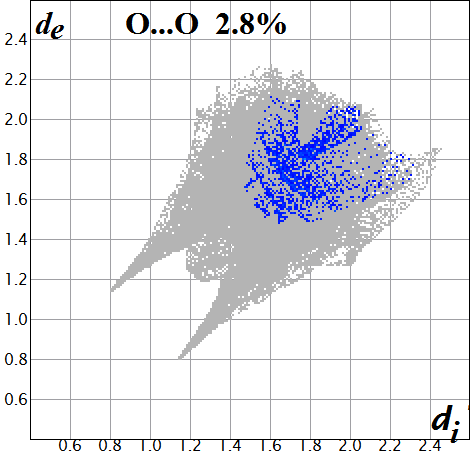 | 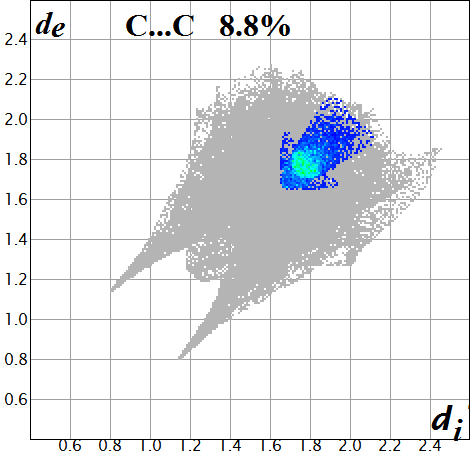 | 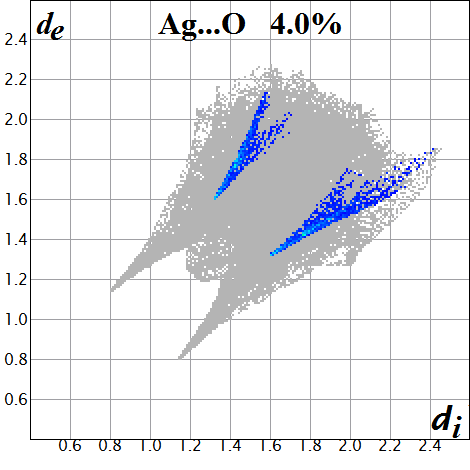 |
| 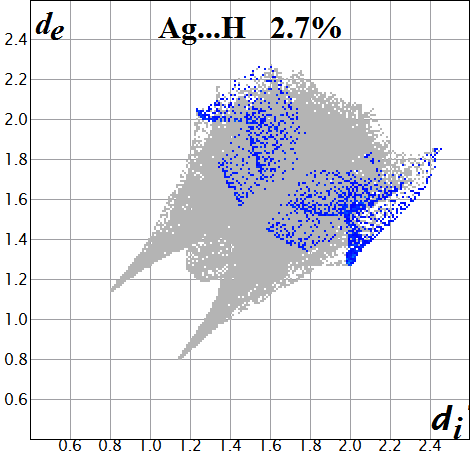 | 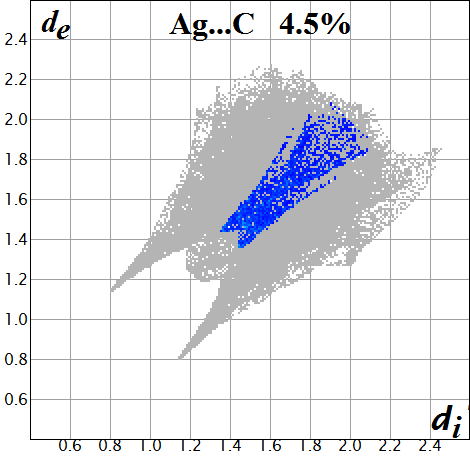 |  |

**Figure S11** The decomposed fingerprint plots of complex **3**.

| 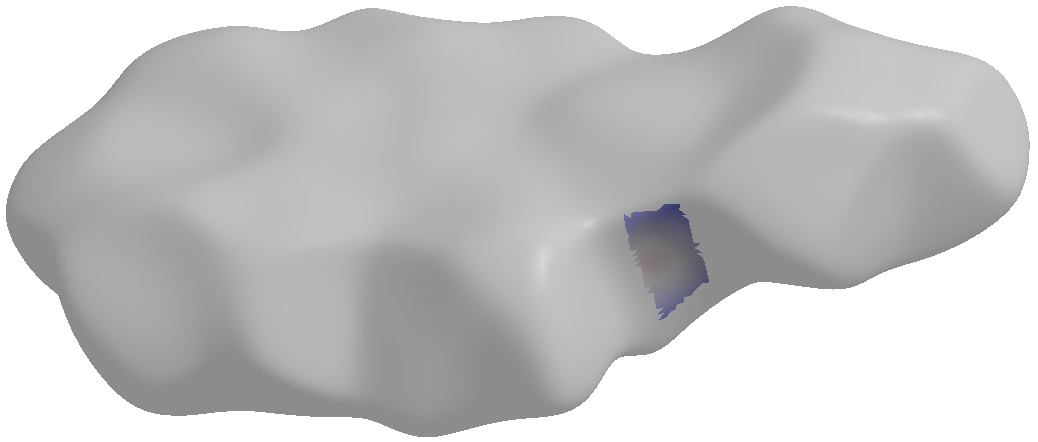 | 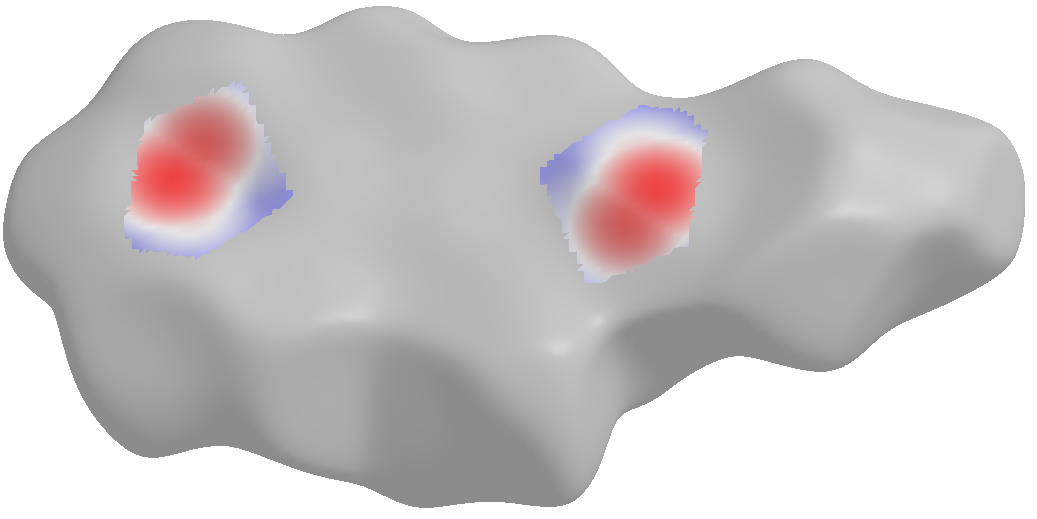 | 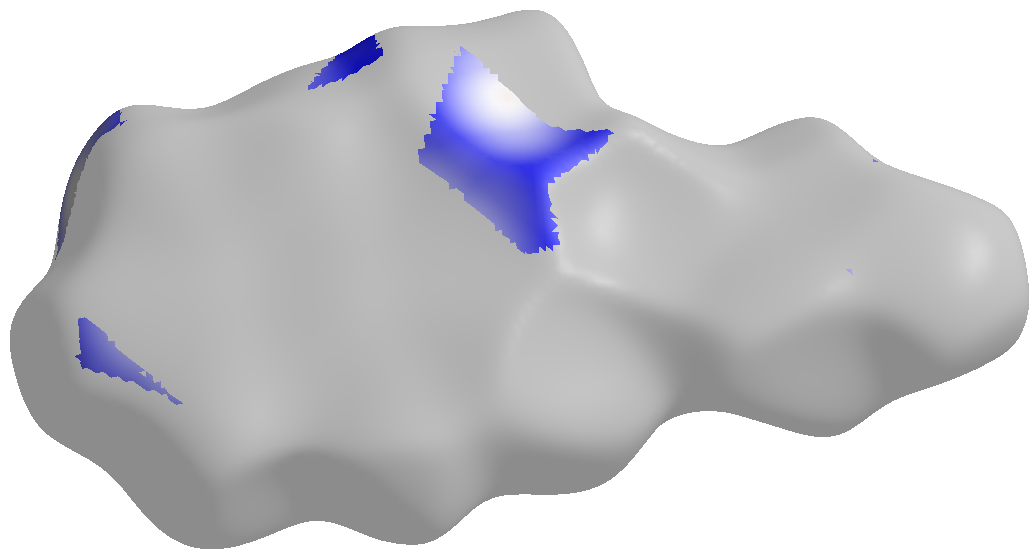 |
| --- | --- | --- |
| **Ag1...O1(3.181Å)** | **Ag-C (2.803-3.291Å)** | **N1...H7(2.607Å)** |
| 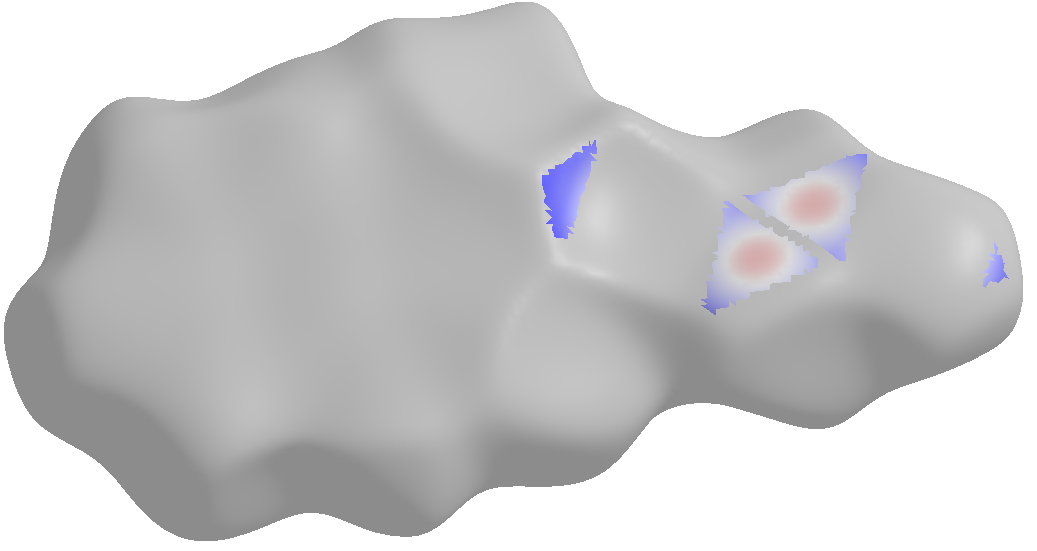 | 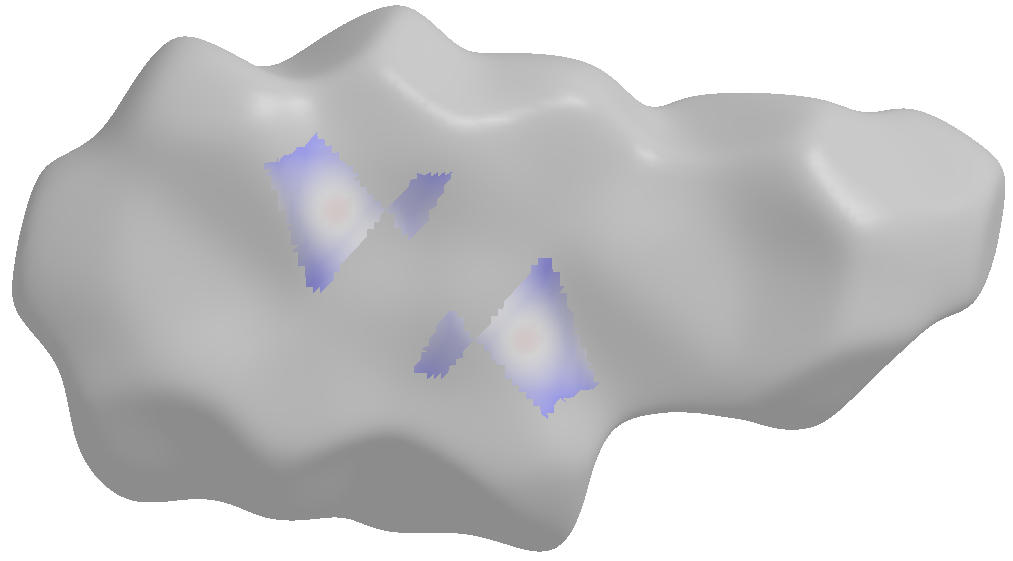 | 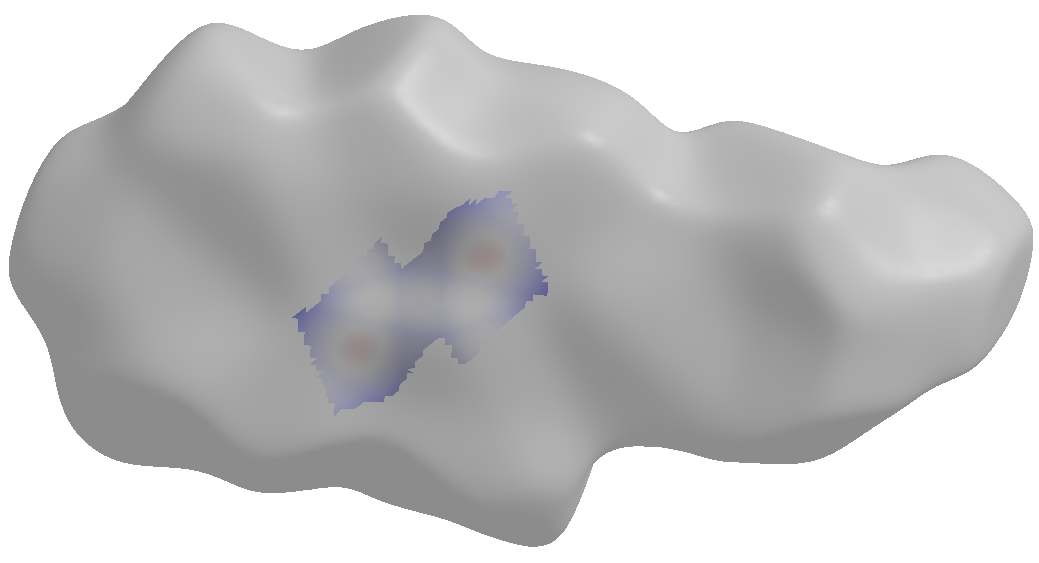 |
| **N4...O3 (2.917Å)** | **N2...C4(3.202Å)** | **C1...C3(3.331Å)** |
| 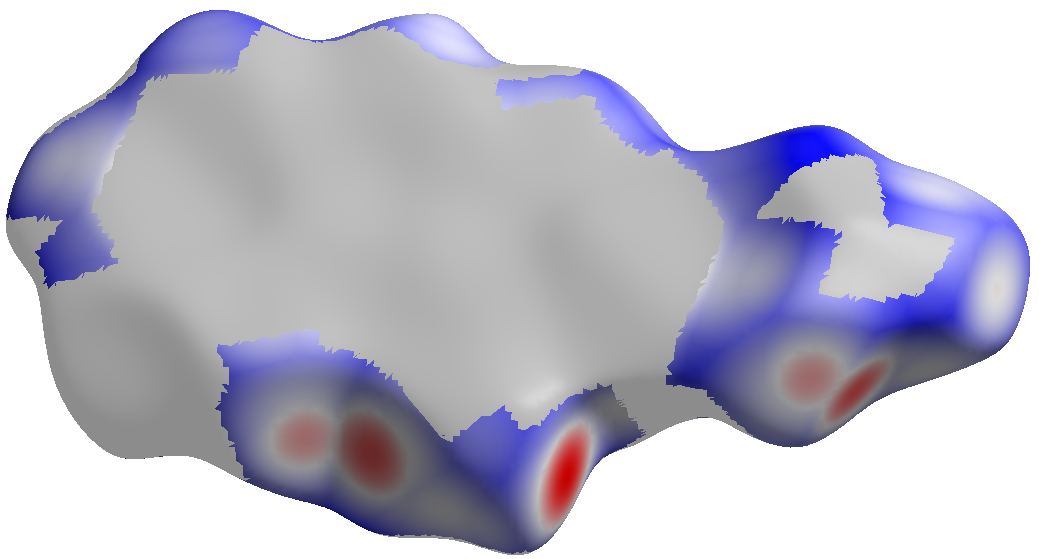 | 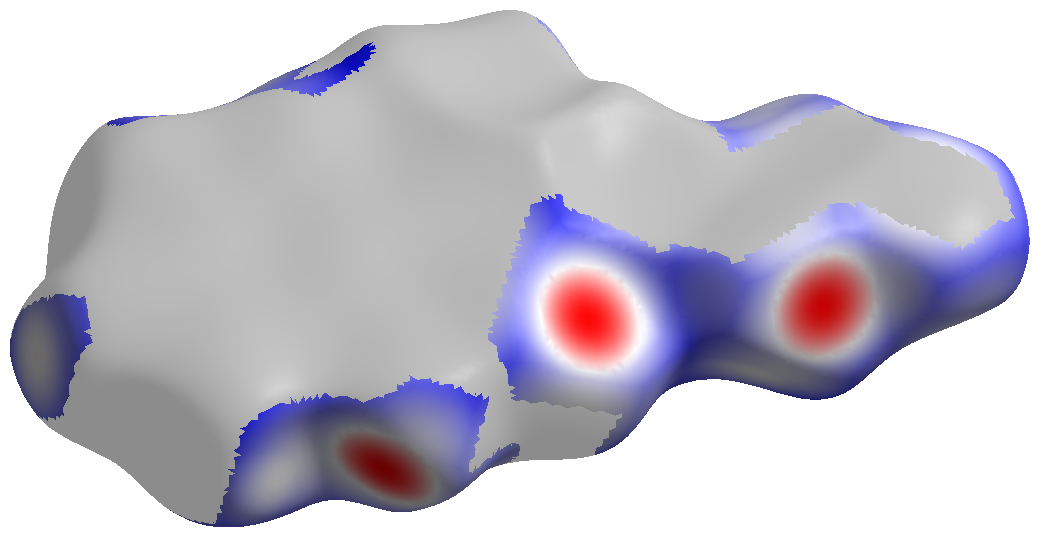 |  |
| **O1...H1B(1.947Å)** | |  |

**Figure S12** The decomposed d_norm_ maps of complex **3**.

| 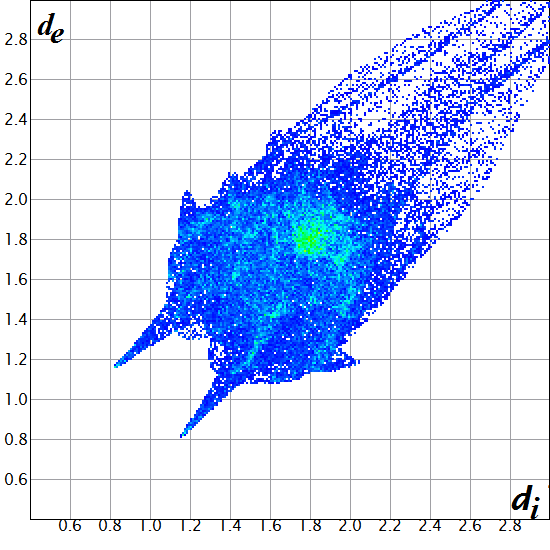 | 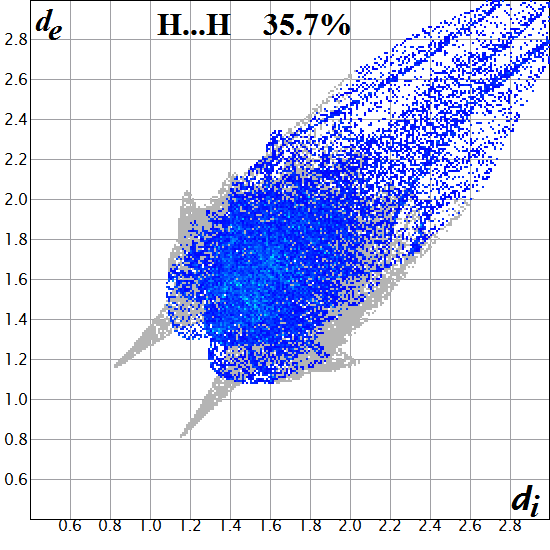 | 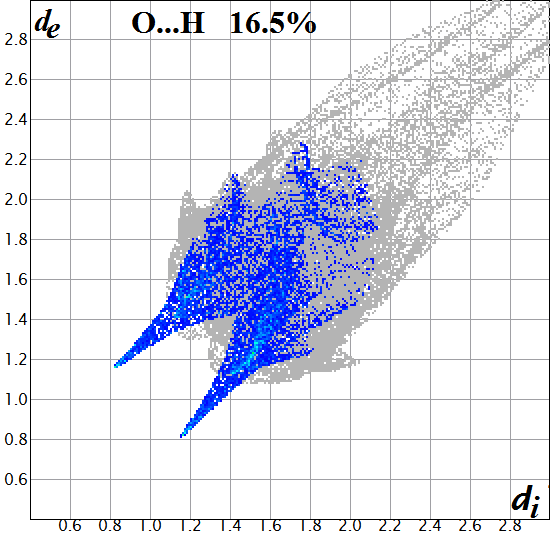 |
| --- | --- | --- |
| 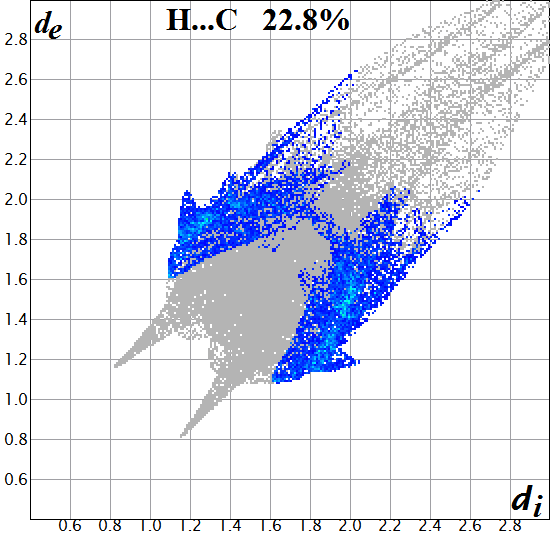 | 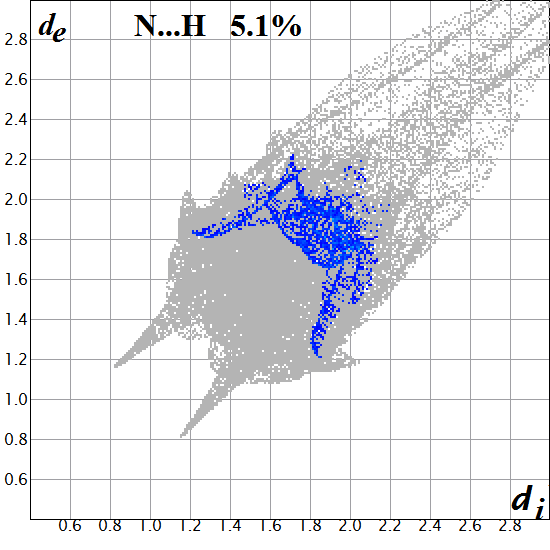 | 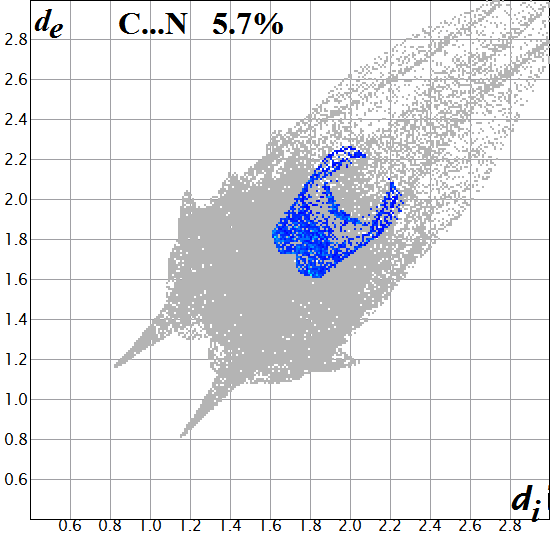 |
| 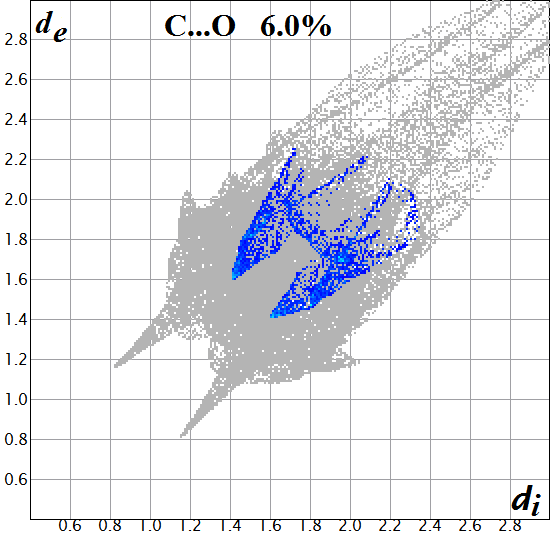 | 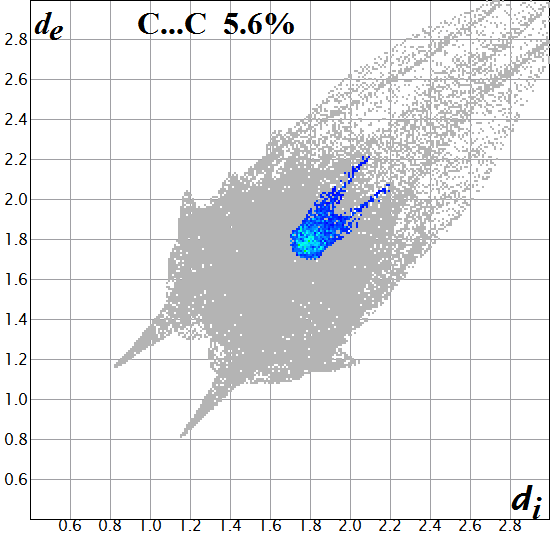 | 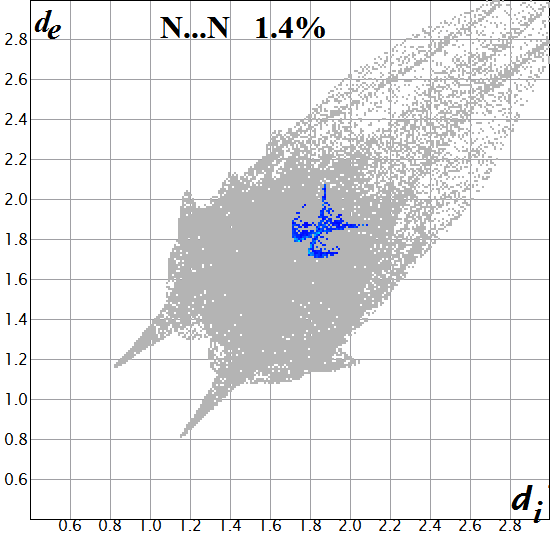 |
| 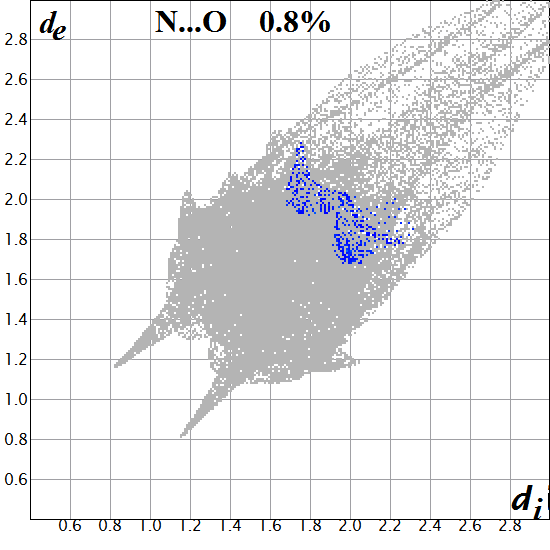 | 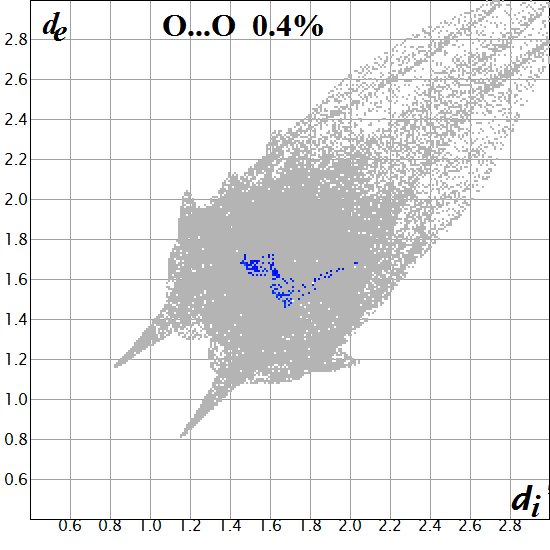 |  |

**Figure S13** The decomposed fingerprint plots of the ligand (**L'**).

| 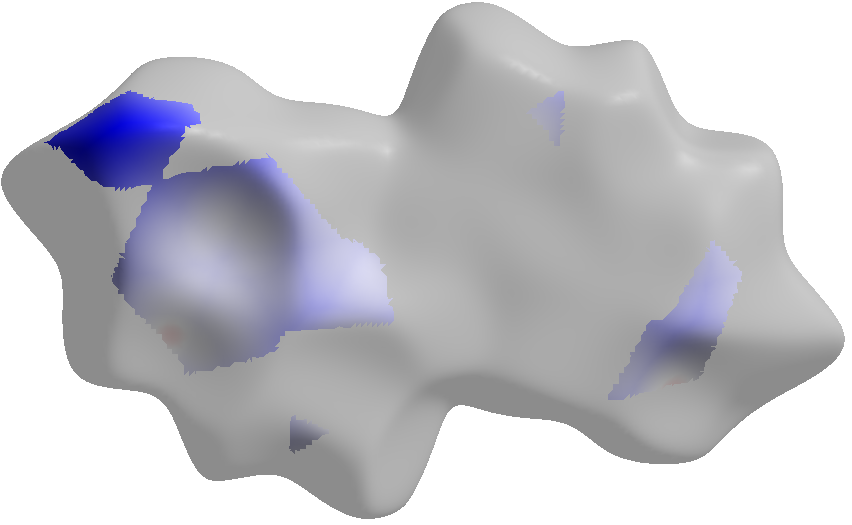 | 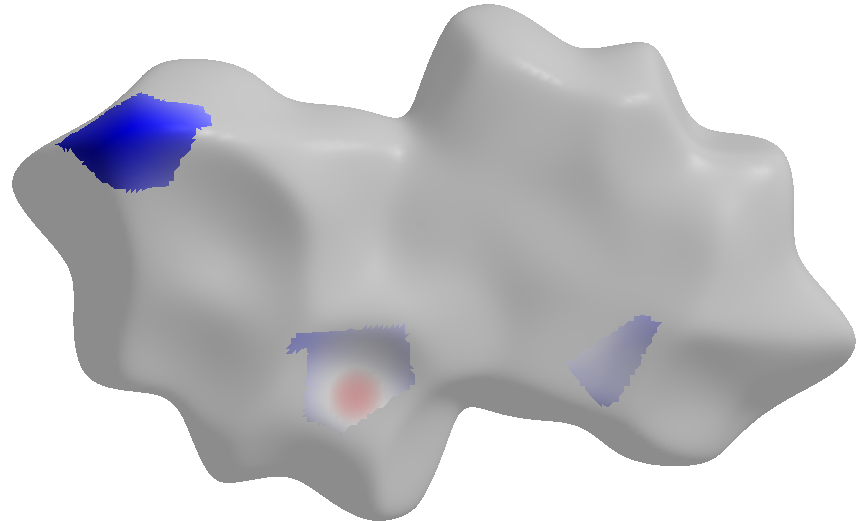 |
| --- | --- |
| **C7…H5 (2.698 Å)** | **C1…O1 (3.018 Å)** |
| 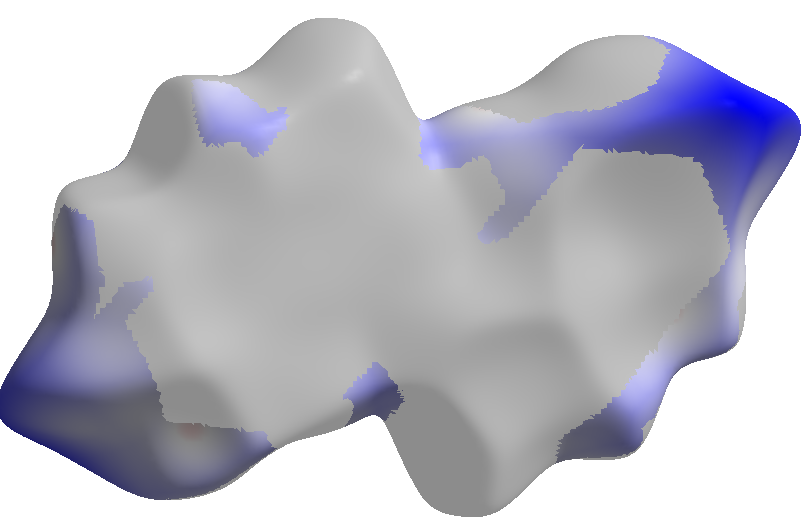 | 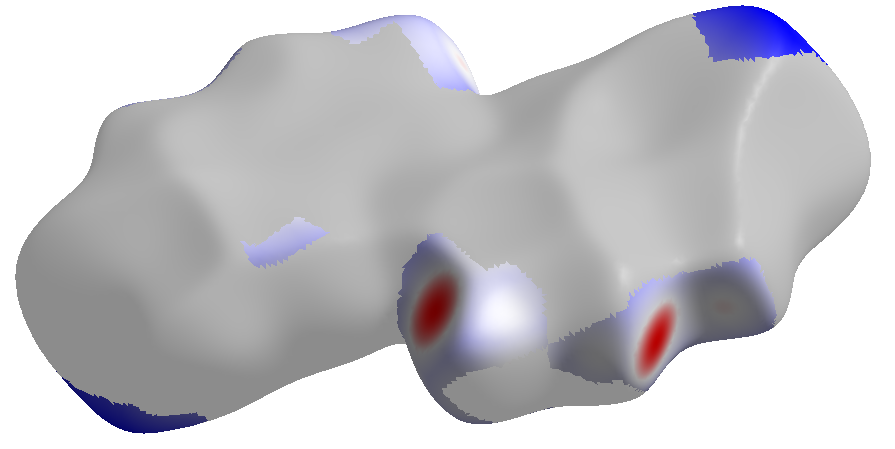 |
| **H5…H7 (2.465 Å)** | **O1…H2 (1.976 Å)** |

**Figure S14** The decomposed d_norm_ maps of the ligand (**L'**).

**Figure S15** The natural orbitals of silver(I) in complex **1**.

**Table S1** Crystallographic data and processing parameters for the complexes **1**- **3** and the pure ligand **L'**

| Compound | **1** | **2** | **3** | **L'** |
| --- | --- | --- | --- | --- |
| CCDC-No | CCDC 1548202 | CCDC 1548204 | CCDC 1548200 | CCDC 1548201 |
| Empirical formula | C_16_H_10_Ag_2_N_6_O_8_ | C_32_H_20_Ag_2_Cl_2_N_8_O_12_ | C_8_H_7_AgN_4_O_4_ | C_16_H_10_N_4_O_2_ |
| Formula weight | 630.04 | 995.2 | 331.05 | 290.28 |
| Temperature | 115(2) K | 118(2) K | 115(2) K | 100(2) K |
| Wavelength | 0.71073 Å | 0.71073 Å | 0.71073 Å | 0.71073 Å |
| Crystal system | Monoclinic | Triclinic | Monoclinic | Trigonal |
| Space group | P 2_1_/c | P -1 | P 2_1_/c | R -3 |
| Unit cell dimensions | a = 15.6591(8) Å | a = 8.9696(2) Å | a = 7.3507(2) Å | a = 24.569(2) Å |
|  | b = 12.7362(7) Å | b = 13.0734(3) Å | b = 12.7515(4) Å | b = 24.569 Å |
|  | c = 9.0375(5) Å | c = 14.7528(3) Å | c = 10.7973(4) Å | c = 5.6130(4) Å |
|  | α = 90° | α= 77.185(6)° | α = 90° | α = 90° |
|  | β = 95.490(2)° | β= 89.162(7)° | β= 107.6510(10)° | β = 90° |
|  | γ = 90° | γ = 84.364(6)° | γ = 90° | γ = 120° |
| Volume | 1794.15(17) Å^3^ | 1678.67(8) Å^3^ | 964.41(5) Å^3^ | 2934.2(5) Å^3^ |
| Z | 4 | 2 | 4 | 9 |
| Density (calculated) | 2.332 Mg/m^3^ | 1.969 Mg/m^3^ | 2.280 Mg/m^3^ | 1.478 Mg/m^3^ |
| Absorption coefficient | 2.249 mm^-1^ | 1.406 mm^-1^ | 2.101 mm^-1^ | 0.102 mm^-1^ |
| F(000) | 1224 | 984 | 648 | 1350 |
| Crystal size [mm^3^] | 0.320 x 0.090 x 0.080 | 0.448 x 0.263 x 0.194 | 0.300 x 0.220 x 0.180 | 0.306 x 0.102 x 0.078 |
| Theta range for data coll. | 2.772 to 25.349° | 2.282 to 25.310° | 2.544 to 25.342° | 2.872 to 25.292° |
| Index ranges | -18<=h<=18 | -10<=h<=10 | -8<=h<=8 | -29<=h<=29 |
|  | -15<=k<=15 | -15<=k<=15 | -15<=k<=15 | -29<=k<=29 |
|  | -10<=l<=10 | -17<=l<=17 | -11<=l<=13 | -6<=l<=6 |
| Reflections collected | 28571 | 27043 | 6238 | 5013 |
| Independent reflections | 3284 [R_(int)_ = 0.0697] | 6098 [R_(int)_ = 0.0311] | 1753 [R(int) = 0.0320] | 1192 [R_(int)_ = 0.0720] |
| Completeness to theta_max_ | 99.8 %, 25.242° | 99.9 %, 25.242° | 99.3 %, 25.242° | 99.9 %, 25.242° |
| Refinement method | All refined with full-matrix least-squares on F^2^ | | | |
| Data / restraints / parameters | 3284 / 0 / 290 | 6098 / 3 / 524 | 1753 / 1 / 157 | 1192 / 0 / 104 |
| Goodness-of-fit on F2 | 1.194 | 1.092 | 1.047 | 1.058 |
| Final R indices [I>2sigma(I)] | R1 = 0.0482 | R1 = 0.0241 | R1 = 0.0379 | R1 = 0.0428 |
|  | wR2 = 0.0923 | wR2 = 0.0509 | wR2 = 0.0944 | wR2 = 0.0851 |
| R indices (all data) | R1 = 0.0647 | R1 = 0.0307 | R1 = 0.0468 | R1 = 0.0702 |
|  | wR2 = 0.0972 | wR2 = 0.0537 | wR2 = 0.0996 | wR2 = 0.0944 |
| Extinction coefficient | 0.0010(2) | 0.00249(18) | n/a | 0.0015(3) |
| Largest diff. peak and hole (e.Å-3) | 2.179 and -0.983 | 0.937 and -0.688 | 1.750 and -0.793 | 0.255 and -0.208 |

**Table S2** The AIM topological parameters of the Ag-N, Ag-O and Ag-C interactions (A…B) in the studied complexes.

| **Complex 1** | | | | | | | | | | | | |
| --- | --- | --- | --- | --- | --- | --- | --- | --- | --- | --- | --- | --- |
| **WB97XD** | | | | | **LC-wPBE** | | | | **B3LYP** | | | |
| **A…B** | **ρ (r)** | **H (r)** | **V(r)/G(r)** | **E_int_** | **ρ (r)** | **H (r)** | **V(r)/G(r)** | **E_int_** | **ρ (r)** | **H (r)** | **V(r)/G(r)** | **E_int_** |
| Ag1-N2 | 0.0539 | -0.0095 | 1.12 | 26.97 | 0.0546 | -0.0101 | 1.13 | 27.09 | 0.0536 | -0.0084 | 1.11 | 26.32 |
| Ag1-N3 | 0.0431 | -0.0051 | 1.09 | 20.08 | 0.0437 | -0.0056 | 1.10 | 20.19 | 0.0431 | -0.0045 | 1.08 | 19.75 |
| Ag1-O3 | 0.035 | -0.0019 | 1.04 | 15.85 | 0.0355 | -0.0022 | 1.04 | 16.05 | 0.0177 | -0.0016 | 1.03 | 15.62 |
| Ag1-O4 | 0.0221 | 0.0003 | 0.99 | 8.64 | 0.0224 | 0.0002 | 0.99 | 8.66 | 0.0227 | 0.0005 | 0.98 | 8.72 |
| Ag1-O2 | 0.0298 | 0.0001 | 1.00 | 12.66 | 0.0301 | -0.0001 | 1.00 | 12.76 | 0.0302 | 0.0002 | 0.99 | 12.75 |
| Ag1-O1 | 0.0234 | 0.001 | 0.96 | 8.96 | 0.0236 | 0.0009 | 0.97 | 9.03 | 0.0238 | 0.0011 | 0.96 | 9.13 |
| Ag2-O5 | 0.031 | -0.0007 | 1.02 | 13.67 | 0.0315 | -0.0009 | 1.02 | 13.78 | 0.0311 | -0.0001 | 1.00 | 13.64 |
| Ag2-O4 | 0.0271 | -0.0004 | 1.01 | 11.13 | 0.0274 | -0.0005 | 1.01 | 11.19 | 0.0271 | 0.0002 | 0.99 | 11.12 |
| Ag2-O6 | 0.0349 | -0.0021 | 1.04 | 15.98 | 0.0356 | -0.0024 | 1.05 | 16.24 | 0.0346 | -0.001 | 1.02 | 15.80 |
| Ag2-O8 | 0.0209 | 0.0008 | 0.97 | 8.14 | 0.0211 | 0.0007 | 0.97 | 8.14 | 0.0212 | 0.0011 | 0.96 | 8.21 |
| Ag2-O6 | 0.0367 | -0.0027 | 1.05 | 16.76 | 0.0373 | -0.003 | 1.06 | 17.01 | 0.0365 | -0.0017 | 1.03 | 16.58 |
| Ag2-O7 | 0.0192 | 0.0011 | 0.96 | 7.20 | 0.0194 | 0.001 | 0.96 | 7.19 | 0.0196 | 0.0012 | 0.95 | 7.32 |
| Ag2-C7 | 0.0222 | -0.0011 | 1.04 | 8.23 | 0.0224 | -0.0011 | 1.04 | 8.28 | 0.0225 | -0.001 | 1.04 | 8.26 |
| **Complex 2** | | | | | | | | | | | | |
| Ag2-N6 | 0.0539 | -0.0095 | 1.12 | 26.88 | 0.0546 | -0.0102 | 1.13 | 27.01 | 0.0537 | -0.0086 | 1.11 | 26.33 |
| Ag2-N3 | 0.0485 | -0.007 | 1.10 | 23.58 | 0.0492 | -0.0076 | 1.11 | 23.70 | 0.0488 | -0.0065 | 1.10 | 23.19 |
| Ag2-O2 | 0.0285 | 0.0004 | 0.99 | 11.84 | 0.0288 | 0.0003 | 0.99 | 11.91 | 0.029 | 0.0005 | 0.99 | 11.99 |
| Ag2-O3 | 0.0278 | 0.0002 | 0.99 | 11.29 | 0.0281 | 0.0001 | 1.00 | 11.37 | 0.0282 | 0.0003 | 0.99 | 11.44 |
| Ag2-O9 | 0.0405 | -0.0032 | 1.06 | 19.17 | 0.0412 | -0.0037 | 1.06 | 19.40 | 0.0406 | -0.0027 | 1.05 | 19.00 |
| Ag1-N1 | 0.0516 | -0.0086 | 1.12 | 24.96 | 0.0524 | -0.0093 | 1.13 | 25.09 | 0.0511 | -0.0077 | 1.11 | 24.42 |
| Ag1-N5 | 0.0545 | -0.0098 | 1.13 | 27.04 | 0.0554 | -0.0107 | 1.14 | 27.23 | 0.0537 | -0.0087 | 1.12 | 26.45 |
| Ag1-O4 | 0.026 | 0.0006 | 0.98 | 10.38 | 0.0263 | 0.0004 | 0.99 | 10.45 | 0.0263 | 0.0006 | 0.98 | 10.51 |
| Ag1-O1 | 0.0236 | 0.0008 | 0.97 | 8.81 | 0.0238 | 0.0007 | 0.97 | 8.87 | 0.0239 | 0.0009 | 0.97 | 8.97 |
| Ag1-O5A | 0.0391 | -0.0026 | 1.05 | 17.92 | 0.0396 | -0.003 | 1.06 | 18.09 | 0.039 | -0.0023 | 1.04 | 17.82 |
| **Complex 3** | | | | | | | | | | | | |
| Ag-N2 | 0.0593 | -0.0107 | 1.11 | 32.78 | 0.0602 | -0.0116 | 1.12 | 32.99 | 0.0582 | -0.0089 | 1.10 | 31.82 |
| Ag1-O1 | 0.0205 | 0.0013 | 0.95 | 7.58 | 0.0207 | 0.0012 | 0.95 | 7.64 | 0.0215 | 0.001 | 0.96 | 7.78 |
| Ag1-O2 | 0.0273 | 0.0005 | 0.99 | 12.62 | 0.0277 | 0.0003 | 0.99 | 12.73 | 0.0279 | 0.0004 | 0.99 | 12.79 |
| Ag1-O3 | 0.0351 | -0.001 | 1.02 | 17.35 | 0.0358 | -0.0014 | 1.03 | 17.58 | 0.0356 | -0.0009 | 1.02 | 17.44 |

ρ(r):electron density; e/a_0_^3^, $H(r)$ total energy density; au, V(r)/G(r): ratio of potential to kinetic energy density and E_int_ (kcal/mol)

**Table S3** The detailed donor-acceptor interactions for the Ag-N, Ag-O and Ag-C interactions of the studied complexes.

| **1** | | | | | **2** | | | | | **3** | | | | |
| --- | --- | --- | --- | --- | --- | --- | --- | --- | --- | --- | --- | --- | --- | --- |
| **NBO_i_** | **NBO_j_** | **B3LYP** | **WB97XD** | **LC-wPBE** | **NBO_i_** | **NBO_j_** | **B3LYP** | **WB97XD** | **LC-wPBE** | **NBO_i_** | **NBO_j_** | **B3LYP** | **WB97XD** | **LC-wPBE** |
| LP(1)N2 | LP*(6)Ag1 | 24.79 | 25.72 | 24.67 | LP(1)O9 | LP*(6)Ag2 | 13.37 | 9.67 | 10.33 | LP(1)N1 | LP*(6)Ag | 37.28 | 39.05 | 38.83 |
| LP(1)N2 | LP*(7)Ag1 |  | 2.62 | 3.93 | LP(1)O9 | LP*(7)Ag2 |  | 9.57 | 9.31 | LP(1)N1 | LP*(7)Ag |  | 20.66 | 23.34 |
| LP(1)N2 | LP*(8)Ag1 |  | 10.63 | 8.76 | LP(1)O9 | LP*(8)Ag2 |  | 10.52 | 11.83 | LP(1)N1 | LP*(8)Ag |  | 2.76 | 3.15 |
| LP(1)N2 | LP*(9)Ag1 |  | 8.81 | 11.3 | LP(1)O9 | LP*(9)Ag2 |  | 0.53 | 0.39 | LP(1)N1 | LP*(9)Ag |  | 0.07 |  |
| LP(1)O1 | LP*(6)Ag1 | 5.33 | 6.31 | 6.72 | LP(3)O9 | LP*(6)Ag2 | 10.7 | 12.38 | 11.5 | LP(1)O1 | LP*(6)Ag | 2.34 | 2.46 | 2.73 |
| LP(1)O1 | LP*(7)Ag1 |  | 4.29 | 7.67 | LP(3)O9 | LP*(7)Ag2 |  | 5.1 | 4.83 | LP(1)O1 | LP*(7)Ag |  | 2.96 | 3.22 |
| LP(1)O1 | LP*(8)Ag1 |  | 5.87 | 4.47 | LP(3)O9 | LP*(8)Ag2 |  | 1.82 | 1.85 | LP(1)O1 | LP*(8)Ag |  | 3.61 | 4.04 |
| LP(1)O1 | LP*(9)Ag1 |  | 4.74 | 3.84 | LP(1)O3 | LP*(6)Ag2 | 5.6 | 5.32 | 4.9 | LP(1)O1 | LP*(9)Ag |  | 0.07 |  |
| LP(2)O1 | LP*(6)Ag1 | 2.46 | 4.18 | 4.02 | LP(1)O3 | LP*(7)Ag2 |  | 6.31 | 7.31 | LP(2)O1 | LP*(6)Ag | 4.24 | 5.8 | 5.55 |
| LP(2)O1 | LP*(7)Ag1 |  | 0.87 | 1.64 | LP(1)O3 | LP*(8)Ag2 |  | 4.59 | 5.69 | LP(2)O1 | LP*(7)Ag |  | 2.21 | 2.47 |
| LP(2)O1 | LP*(8)Ag1 |  | 1.95 | 1.58 | LP(1)O3 | LP*(9)Ag2 |  | 4.89 | 3.99 | LP(2)O1 | LP*(8)Ag |  | 2 | 2.09 |
| LP(2)O1 | LP*(9)Ag1 |  | 0.52 | 0.33 | LP(2)O3 | LP*(6)Ag2 | 3.78 | 4.99 | 4.88 | LP(1)O2 | LP*(6)Ag | 3.67 | 3.33 | 3.55 |
| LP(1)O3 | LP*(6)Ag1 | 8.12 | 6.31 | 6.64 | LP(2)O3 | LP*(7)Ag2 |  | 2.71 | 3.3 | LP(1)O2 | LP*(7)Ag |  | 4.55 | 4.69 |
| LP(1)O3 | LP*(7)Ag1 |  | 6.4 | 4.7 | LP(2)O3 | LP*(8)Ag2 |  | 1.12 | 1.26 | LP(1)O2 | LP*(8)Ag |  | 0.38 | 0.55 |
| LP(1)O3 | LP*(8)Ag1 |  | 5.67 | 7.91 | LP(2)O3 | LP*(9)Ag2 |  | 1.26 | 1.02 | LP(2)O2 | LP*(6)Ag | 19.78 | 25.18 | 26.68 |
| LP(1)O3 | LP*(9)Ag1 |  | 2.78 | 2.45 | LP(1)N6 | LP*(6)Ag2 | 24.24 | 27.57 | 27.07 | LP(2)O2 | LP*(7)Ag |  | 3.27 | 3.6 |
| LP(2)O3 | LP*(6)Ag1 | 10.52 | 17.48 | 18.86 | LP(1)N6 | LP*(7)Ag2 |  | 13.72 | 14.91 | LP(2)O2 | LP*(8)Ag |  | 1.92 | 2.5 |
| LP(2)O3 | LP*(7)Ag1 |  | 5.09 | 3.58 | LP(1)N6 | LP*(8)Ag2 |  | 0.18 | 0.51 | LP(3)O2 | LP*(6)Ag | 0.18 | 0.22 | 0.2 |
| LP(2)O3 | LP*(8)Ag1 |  | 4.76 | 7.26 | LP(1)N6 | LP*(9)Ag2 |  | 4.59 | 5.4 | LP(3)O2 | LP*(8)Ag |  | 0.11 | 0.12 |
| LP(2)O3 | LP*(9)Ag1 |  | 1.47 | 1.27 | LP(1)O2 | LP*(6)Ag2 | 5.67 | 7.25 | 7.04 | LP(3)O2 | LP*(9)Ag |  | 1.41 | 1.36 |
| LP(1)O4 | LP*(6)Ag1 | 6.78 | 6.51 | 7.1 | LP(1)O2 | LP*(7)Ag2 |  | 1.91 | 2.29 | LP(1)O3 | LP*(6)Ag | 4.52 | 4.46 | 4.75 |
| LP(1)O4 | LP*(7)Ag1 |  | 0.26 | 1.97 | LP(1)O2 | LP*(8)Ag2 |  | 8.51 | 9.71 | LP(1)O3 | LP*(7)Ag |  | 2.09 | 2.16 |
| LP(1)O4 | P*(8)Ag1 |  | 12.88 | 12.47 | LP(1)O2 | LP*(9)Ag2 |  | 3.25 | 2.58 | LP(1)O3 | LP*(8)Ag |  | 4.11 | 4.17 |
| LP(1)O4 | LP*(9)Ag1 |  | 0.54 | 0.38 | LP(2)O2 | LP*(6)Ag2 | 3.19 | 4.53 | 4.53 | LP(1)O3 | LP*(9)Ag |  | 0.17 | 0.15 |
| LP(2)O4 | LP*(6)Ag1 | 4.01 | 5.95 | 6.05 | LP(2)O2 | LP*(7)Ag2 |  | 0.53 | 0.5 | LP(2)O3 | LP*(6)Ag | 25.01 | 33.56 | 35.95 |
| LP(2)O4 | LP*(7)Ag1 |  | 0.25 | 0.8 | LP(2)O2 | LP*(8)Ag2 |  | 2.2 | 2.54 | LP(2)O3 | LP*(7)Ag |  | 0.57 | 0.71 |
| LP(2)O4 | LP*(8)Ag1 |  | 2.12 | 1.88 | LP(2)O2 | LP*(9)Ag2 |  | 0.22 | 0.1 | LP(2)O3 | LP*(8)Ag |  | 6.52 | 6.72 |
| LP(2)O4 | LP*(9)Ag1 |  | 0.51 | 0.41 | LP(1)N3 | LP*(6)Ag2 | 20.47 | 22.59 | 21.88 | LP(2)O3 | LP*(9)Ag |  | 0.14 | 0.08 |
| LP(1)O4 | LP*(6)Ag2 | 9.18 | 7.94 | 8.25 | LP(1)N3 | LP*(7)Ag2 |  | 0.3 | 0.62 | LP(3)O3 | LP*(6)Ag | 0.22 | 1.98 | 0.27 |
| LP(1)O4 | LP*(7)Ag2 |  | 4.66 | 5.1 | LP(1)N3 | LP*(8)Ag2 |  | 9.25 | 8.4 | LP(3)O3 | LP*(9)Ag |  | 1.98 | 1.95 |
| LP(1)O4 | LP*(8)Ag2 |  | 8.71 | 9.78 | LP(1)N3 | LP*(9)Ag2 |  | 10.52 | 12.32 |  |  |  |  |  |
| LP(1)O4 | LP*(9)Ag2 |  | 4.86 | 4.89 | LP(1)O1 | LP*(6)Ag1 | 4.79 | 5.95 | 6.31 |  |  |  |  |  |
| LP(2)O4 | LP*(6)Ag2 | 7.41 | 7.39 | 7.02 | LP(1)O1 | LP*(7)Ag1 |  | 3.18 | 5.31 |  |  |  |  |  |
| LP(2)O4 | LP*(7)Ag2 |  | 0.76 | 0.88 | LP(1)O1 | LP*(8)Ag1 |  | 8.69 | 7.49 |  |  |  |  |  |
| LP(2)O4 | LP*(8)Ag2 |  | 0.96 | 1.24 | LP(1)O1 | LP*(9)Ag1 |  | 2.36 | 2.38 |  |  |  |  |  |
| LP(2)O4 | LP*(9)Ag2 |  | 0.18 | 0.18 | LP(2)O1 | LP*(6)Ag1 | 2.09 | 3.22 | 3.18 |  |  |  |  |  |
| LP(1)O5 | LP*(6)Ag2 | 8.26 | 4.75 | 4.4 | LP(2)O1 | LP*(7)Ag1 |  | 0.26 | 0.55 |  |  |  |  |  |
| LP(1)O5 | LP*(7)Ag2 |  | 10.47 | 10.84 | LP(2)O1 | LP*(8)Ag1 |  | 1.46 | 1.29 |  |  |  |  |  |
| LP(1)O5 | LP*(8)Ag2 |  | 5.72 | 5.56 | LP(2)O1 | LP*(9)Ag1 |  | 0.15 | 0.11 |  |  |  |  |  |
| LP(1)O5 | LP*(9)Ag2 |  | 3.04 | 3.41 | LP(1)O5A | LP*(6)Ag1 | 11.31 | 11.67 | 12.81 |  |  |  |  |  |
| LP(2)O5 | LP*(6)Ag2 | 12.06 | 16.35 | 16.08 | LP(1)O5A | LP*(7)Ag1 |  | 17.51 | 18.02 |  |  |  |  |  |
| LP(2)O5 | LP*(7)Ag2 |  | 10.53 | 12.04 | LP(1)O5A | LP*(8)Ag1 |  | 0.08 | 1.07 |  |  |  |  |  |
| LP(2)O5 | LP*( 8)Ag2 |  | 5.42 | 6.03 | LP(1)O5A | LP*(9)Ag1 |  | 3.59 | 3.07 |  |  |  |  |  |
| LP(2)O5 | LP*(9)Ag2 |  | 5.11 | 5.85 | LP(3)O5A | LP*(6)Ag1 | 6.62 | 8.67 | 8.28 |  |  |  |  |  |
| LP(1)O6 | LP*(6)Ag2 | 9.25 | 5.11 | 4.75 | LP(3)O5A | LP*(7)Ag1 |  | 2.59 | 2.65 |  |  |  |  |  |
| LP(1)O6 | LP*(7)Ag2 |  | 8.78 | 8.56 | LP(3)O5A | LP*(8)Ag1 |  | 0.36 | 0.1 |  |  |  |  |  |
| LP(1)O6 | LP*(8)Ag2 |  | 7.32 | 6.91 | LP(3)O5A | LP*(9)Ag1 |  | 1.27 | 1.01 |  |  |  |  |  |
| LP(2)O6 | LP*(6)Ag2 | 18.67 | 26.93 | 27.66 | LP(1)O4 | LP*(6)Ag1 | 5.53 | 6.14 | 6.39 |  |  |  |  |  |
| LP(2)O6 | LP*(7)Ag2 |  | 14.85 | 16.21 | LP(1)O4 | LP*(8)Ag1 |  | 11.1 | 11.95 |  |  |  |  |  |
| LP(2)O6 | LP*(8)Ag2 |  | 13.06 | 14.07 | LP(1)O4 | LP*(9)Ag1 |  | 4.08 | 4.12 |  |  |  |  |  |
| LP(1)O7 | LP*(6)Ag2 | 6.09 | 5.96 | 6.34 | LP(2)O4 | LP*(6)Ag1 | 2.74 | 3.96 | 4.07 |  |  |  |  |  |
| LP(1)O7 | LP*(7)Ag2 |  | 0.53 | 0.68 | LP(2)O4 | LP*(8)Ag |  | 1.95 | 2.08 |  |  |  |  |  |
| LP(1)O7 | LP*(8)Ag2 |  | 12.75 | 12.7 | LP(2)O4 | LP*(9)Ag1 |  | 0.42 | 0.45 |  |  |  |  |  |
| LP(1)O7 | LP*(9)Ag2 |  | 0.09 | 0.15 | LP(1)N1 | LP*(6)Ag1 | 17.67 | 19.66 | 18.83 |  |  |  |  |  |
| LP(2)O7 | LP*(6)Ag2 | 7.12 | 11.86 | 12.47 | LP(1)N1 | LP*(7)Ag1 |  | 2.09 | 0.71 |  |  |  |  |  |
| LP(2)O7 | LP*(7)Ag2 |  | 0.58 | 0.71 | LP(1)N1 | LP*(8)Ag1 |  | 7.28 | 8.26 |  |  |  |  |  |
| LP(2)O7 | LP*(8)Ag2 |  | 11.57 | 12.19 | LP(1)N1 | LP*(9)Ag1 |  | 5.83 | 7.2 |  |  |  |  |  |
| LP(1)N3 | LP*(6)Ag1 | 19.13 | 23.62 | 23.41 | LP(1)N5 | LP*(6)Ag1 | 20.21 | 23.01 | 22.33 |  |  |  |  |  |
| LP(1)N3 | LP*(7)Ag1 |  | 8.07 | 5.72 | LP(1)N5 | LP*(7)Ag1 |  | 11.02 | 11.25 |  |  |  |  |  |
| LP(1)N3 | LP*(8)Ag1 |  | 1.6 | 4.09 | LP(1)N5 | LP*(8)Ag1 |  | 0.85 | 2.24 |  |  |  |  |  |
| LP(1)N3 | LP*(9)Ag1 |  | 7.97 | 8.91 | LP(1)N5 | LP*(9)Ag 1 |  | 0.11 | 0.12 |  |  |  |  |  |
| LP(1)O2 | LP*(6)Ag1 | 6.94 | 6.78 | 6.88 |  |  |  |  |  |  |  |  |  |  |
| LP(1)O2 | LP*(7)Ag1 |  | 13.06 | 15.13 |  |  |  |  |  |  |  |  |  |  |
| LP(1)O2 | LP*(8)Ag1 |  | 0.06 | 0.26 |  |  |  |  |  |  |  |  |  |  |
| LP(1)O2 | LP*(9)Ag1 |  | 4.39 | 3.59 |  |  |  |  |  |  |  |  |  |  |
| LP(2)O2 | LP*(6)Ag1 | 3.45 | 5.18 | 5.15 |  |  |  |  |  |  |  |  |  |  |
| LP(2)O2 | LP*(7)Ag1 |  | 3.92 | 4.61 |  |  |  |  |  |  |  |  |  |  |
| LP(2)O2 | LP*(9)Ag1 |  | 0.66 | 0.51 |  |  |  |  |  |  |  |  |  |  |
| LP(1)O6 | LP*(6)Ag2 | 8.66 | 5.23 | 4.79 |  |  |  |  |  |  |  |  |  |  |
| LP(1)O6 | LP*(7)Ag2 |  | 12.41 | 12.13 |  |  |  |  |  |  |  |  |  |  |
| LP(1)O6 | LP*(9)Ag2 |  | 3.48 | 3.57 |  |  |  |  |  |  |  |  |  |  |
| LP(2)O6 | LP*(6)Ag2 | 19.4 | 26.99 | 27.58 |  |  |  |  |  |  |  |  |  |  |
| LP(2)O6 | LP*(7)Ag2 |  | 15.08 | 17.03 |  |  |  |  |  |  |  |  |  |  |
| LP(2)O6 | LP*(9)Ag2 |  | 7.09 | 7.9 |  |  |  |  |  |  |  |  |  |  |
| LP(1)O8 | LP*(6)Ag2 | 6.97 | 5.59 | 5.89 |  |  |  |  |  |  |  |  |  |  |
| LP(1)O8 | LP*(7)Ag 2 |  | 7.16 | 7.22 |  |  |  |  |  |  |  |  |  |  |
| LP(1)O8 | LP*(8)Ag2 |  | 1.37 | 1.77 |  |  |  |  |  |  |  |  |  |  |
| LP(1)O8 | LP*(9)Ag2 |  | 2.87 | 2.93 |  |  |  |  |  |  |  |  |  |  |
| LP(2)O8 | LP*(6)Ag2 | 10.09 | 14.16 | 14.64 |  |  |  |  |  |  |  |  |  |  |
| LP(2)O8 | LP*(7)Ag2 |  | 5.26 | 5.97 |  |  |  |  |  |  |  |  |  |  |
| LP(2)O8 | LP*(8)Ag2 |  | 1.28 | 1.91 |  |  |  |  |  |  |  |  |  |  |
| LP(2)O8 | LP*(9)Ag2 |  | 1.94 | 2.13 |  |  |  |  |  |  |  |  |  |  |
| BD(2)C7-C6 | LP*(6)Ag2 | 10.81 | 9.95 | 8.41 |  |  |  |  |  |  |  |  |  |  |
| BD(2)C7-C6 | LP*(7)Ag2 |  | 0.97 | 0.96 |  |  |  |  |  |  |  |  |  |  |
| BD(2)C7-C6 | LP*(8)Ag2 |  | 0.22 | 0.23 |  |  |  |  |  |  |  |  |  |  |
| BD(2)C7-C6 | LP*( 9)Ag2 |  | 7.17 | 7.03 |  |  |  |  |  |  |  |  |  |  |
|  |  |  |  |  |  |  |  |  |  |  |  |  |  |  |

**Table S4** The net donor (NBO_i_)-acceptor (NBO_j_) interactionenergies included in the Ag-O, Ag-N and Ag-C interactions.

| **NBO_I_**→**NBO_J_** | **B3LYP** | **WB97XD** | **LC-wPBE** |
| --- | --- | --- | --- |
| **Complex 1** |  |  |  |
| LPN2→LP*Ag1 | 24.79 | 47.78 | 48.66 |
| LPN3→LP*Ag1 | 19.13 | 41.26 | 42.13 |
| LPO1→LP*Ag1 | 7.79 | 28.73 | 30.27 |
| LPO2→LP*Ag1 | 10.39 | 34.05 | 36.13 |
| LPO3→LP*Ag1 | 18.64 | 49.96 | 52.67 |
| LPO4→LP*Ag1 | 10.79 | 29.02 | 31.06 |
| LPO4→LP*Ag2 | 16.59 | 35.46 | 37.34 |
| LPO5→LP*Ag2 | 20.32 | 61.39 | 64.21 |
| LPO6→LP*Ag2 | 27.92 | 76.05 | 78.16 |
| LPO6#2→LP*Ag2 | 28.06 | 70.28 | 73.00 |
| LPO7→LP*Ag2 | 13.21 | 43.34 | 45.24 |
| LPO8→LP*Ag2 | 17.06 | 39.63 | 42.46 |
| πC7-C6→LP*Ag2 | 10.81 | 18.31 | 16.63 |
| **Complex 2** |  |  |  |
| LPN1→LP*Ag1 | 17.67 | 34.86 | 35.00 |
| LPN5→LP*Ag1 | 20.21 | 34.99 | 35.94 |
| LPO1→LP*Ag1 | 6.88 | 25.27 | 26.62 |
| LPO4→LP*Ag1 | 8.27 | 27.65 | 29.06 |
| LPO5A→LP*Ag1 | 17.93 | 45.74 | 47.01 |
| LPN3→LP*Ag2 | 20.47 | 42.66 | 43.22 |
| LPN6→LP*Ag2 | 24.24 | 46.06 | 47.89 |
| LPO2→LP*Ag2 | 8.86 | 28.40 | 29.29 |
| LPO3→LP*Ag2 | 9.38 | 31.19 | 32.35 |
| LPO9→LP*Ag2 | 24.07 | 49.59 | 50.04 |
| **Complex 3** |  |  |  |
| LPN2→LP*Ag | 37.28 | 62.54 | 65.32 |
| LPO1→LP*Ag | 6.58 | 19.11 | 20.10 |
| LPO2→LP*Ag | 23.63 | 40.37 | 43.25 |
| LPO3→LP*Ag | 29.75 | 55.58 | 56.91 |

| **Table S5** The occupancy and energies of the different natural orbitals included in the Ag-N, Ag-O and Ag-C interactions of the studied complexes. | | | | | | | | | | | | | | | | | | | | |
| --- | --- | --- | --- | --- | --- | --- | --- | --- | --- | --- | --- | --- | --- | --- | --- | --- | --- | --- | --- | --- |
| **Complex 1** | | | | | | | **Complex 2** | | | | | | | **Complex 3** | | | | | | |
| **NBO** | **B3LYP** | | **WB97XD** | | **LC-wPBE** | | **NBO** | **B3LYP** | | **WB97XD** | | **LC-wPBE** | | **NBO** | **B3LYP** | | **WB97XD** | | **LC-wPBE** | |
| LP(1) N2 | 1.840 | -0.379 | 1.834 | -0.453 | 1.844 | -0.484 | LP(1)O9 | 1.965 | -0.782 | 1.939 | -0.832 | 1.936 | -0.847 | LP*(6)Ag | 0.251 | 0.031 | 0.218 | 0.131 | 0.197 | 0.188 |
| LP(1) O1 | 1.969 | -0.668 | 1.949 | -0.745 | 1.948 | -0.765 | LP(3)O9 | 1.848 | -0.294 | 1.847 | -0.383 | 1.851 | -0.413 | LP*(7)Ag |  |  | 0.055 | 0.460 | 0.058 | 0.483 |
| LP(2) O1 | 1.843 | -0.235 | 1.845 | -0.319 | 1.850 | -0.353 | LP(1)O3 | 1.968 | -0.719 | 1.949 | -0.783 | 1.947 | -0.802 | LP*(8)Ag |  |  | 0.039 | 0.217 | 0.039 | 0.229 |
| LP(1)O3 | 1.969 | -0.676 | 1.949 | -0.681 | 1.947 | -0.692 | LP(2)O3 | 1.852 | -0.289 | 1.854 | -0.371 | 1.859 | -0.405 | LP*(9)Ag |  |  | 0.011 | 0.144 | 0.010 | 0.160 |
| LP(2)O3 | 1.888 | -0.234 | 1.882 | -0.376 | 1.885 | -0.415 | LP(1)N6 | 1.845 | -0.432 | 1.836 | -0.497 | 1.846 | -0.527 | LP(1) N1 | 1.867 | -0.466 | 1.863 | -0.538 | 1.870 | -0.571 |
| LP(1)O4 | 1.959 | -0.709 | 1.917 | -0.777 | 1.914 | -0.792 | LP(1)O2 | 1.967 | -0.733 | 1.949 | -0.805 | 1.948 | -0.825 | LP(1) O1 | 1.974 | -0.747 | 1.966 | -0.830 | 1.966 | -0.850 |
| LP(2)O4 | 1.888 | -0.207 | 1.883 | -0.272 | 1.887 | -0.306 | LP(2)O2 | 1.842 | -0.303 | 1.844 | -0.381 | 1.848 | -0.414 | LP(2) O1 | 1.843 | -0.301 | 1.843 | -0.378 | 1.848 | -0.412 |
| LP(1)O5 | 1.971 | -0.688 | 1.952 | -0.691 | 1.951 | -0.704 | LP(1)N3 | 1.856 | -0.456 | 1.845 | -0.525 | 1.855 | -0.556 | LP (1)O2 | 1.977 | -0.720 | 1.969 | -0.780 | 1.968 | -0.792 |
| LP(2)O5 | 1.895 | -0.226 | 1.886 | -0.357 | 1.887 | -0.395 | LP(1)O1 | 1.967 | -0.744 | 1.948 | -0.821 | 1.947 | -0.842 | LP(2)O2 | 1.863 | -0.286 | 1.862 | -0.391 | 1.868 | -0.428 |
| LP(1)O6 | 1.971 | -0.616 | 1.953 | -0.580 | 1.952 | -0.586 | LP(2)O1 | 1.854 | -0.309 | 1.857 | -0.391 | 1.861 | -0.425 | LP(3)O2 | 1.652 | -0.236 | 1.657 | -0.294 | 1.660 | -0.320 |
| LP(2)O6 | 1.896 | -0.206 | 1.884 | -0.374 | 1.886 | -0.417 | LP(1)O5A | 1.964 | -0.819 | 1.938 | -0.892 | 1.936 | -0.908 | LP(1)O3 | 1.976 | -0.710 | 1.967 | -0.766 | 1.967 | -0.776 |
| LP(1)O7 | 1.973 | -0.644 | 1.957 | -0.674 | 1.956 | -0.687 | LP(3)O5A | 1.854 | -0.316 | 1.855 | -0.400 | 1.859 | -0.430 | LP(2)O3 | 1.864 | -0.293 | 1.864 | -0.404 | 1.869 | -0.442 |
| LP(2)O7 | 1.891 | -0.160 | 1.882 | -0.265 | 1.884 | -0.301 | LP(1)O4 | 1.966 | -0.748 | 1.948 | -0.825 | 1.947 | -0.846 | LP(3)O3 | 1.654 | -0.233 | 1.662 | -0.292 | 1.665 | -0.318 |
| LP(1)N3 | 1.847 | -0.377 | 1.837 | -0.448 | 1.847 | -0.479 | LP(2)O4 | 1.851 | -0.322 | 1.854 | -0.406 | 1.859 | -0.440 |  |  |  |  |  |  |  |
| LP(1)O2 | 1.967 | -0.669 | 1.947 | -0.741 | 1.946 | -0.759 | LP(1)N1 | 1.850 | -0.461 | 1.848 | -0.535 | 1.859 | -0.567 |  |  |  |  |  |  |  |
| LP(2)O2 | 1.852 | -0.241 | 1.854 | -0.329 | 1.859 | -0.363 | LP(1)N5 | 1.850 | -0.461 | 1.849 | -0.539 | 1.859 | -0.571 |  |  |  |  |  |  |  |
| LP(1)O6 | 1.973 | -0.601 | 1.955 | -0.578 | 1.954 | -0.583 | LP*(6)Ag2 | 0.201 | 0.452 | 0.176 | 0.638 | 0.162 | 0.798 |  |  |  |  |  |  |  |
| LP(2)O6 | 1.889 | -0.199 | 1.880 | -0.354 | 1.883 | -0.397 | LP*(7)Ag2 |  |  | 0.082 | 0.321 | 0.084 | 0.349 |  |  |  |  |  |  |  |
| LP(1)O8 | 1.970 | -0.631 | 1.953 | -0.654 | 1.952 | -0.666 | LP*(8)Ag2 |  |  | 0.071 | 0.277 | 0.072 | 0.286 |  |  |  |  |  |  |  |
| LP(2) O8 | 1.886 | -0.162 | 1.878 | -0.272 | 1.880 | -0.309 | LP*(9)Ag2 |  |  | 0.065 | 0.277 | 0.066 | 0.286 |  |  |  |  |  |  |  |
| LP*(6)Ag2 | 0.235 | 0.531 | 0.202 | 0.721 | 0.190 | 0.731 | LP*(6)Ag1 | 0.199 | 0.187 | 0.178 | 0.252 | 0.164 | 0.286 |  |  |  |  |  |  |  |
| LP*(7)Ag2 |  |  | 0.107 | 0.465 | 0.110 | 0.475 | LP*( 7)Ag1 |  |  | 0.082 | 0.286 | 0.084 | 0.289 |  |  |  |  |  |  |  |
| LP*(8)Ag2 |  |  | 0.093 | 0.417 | 0.095 | 0.428 | LP*( 8)Ag1 |  |  | 0.080 | 0.231 | 0.082 | 0.248 |  |  |  |  |  |  |  |
| LP*(9)Ag2 |  |  | 0.069 | 0.515 | 0.070 | 0.521 | LP*(9)Ag1 |  |  | 0.064 | 0.233 | 0.064 | 0.260 |  |  |  |  |  |  |  |
| LP*( 6)Ag1 | 0.207 | 0.561 | 0.192 | 0.652 | 0.180 | 0.645 |  |  |  |  |  |  |  |  |  |  |  |  |  |  |
| LP*( 7)Ag1 |  |  | 0.083 | 0.343 | 0.085 | 0.357 |  |  |  |  |  |  |  |  |  |  |  |  |  |  |
| LP*( 8)Ag1 |  |  | 0.082 | 0.362 | 0.083 | 0.366 |  |  |  |  |  |  |  |  |  |  |  |  |  |  |
| LP*(9)Ag1 |  |  | 0.074 | 0.341 | 0.075 | 0.355 |  |  |  |  |  |  |  |  |  |  |  |  |  |  |

The energy of the free Ag(I) natural orbitals are 4.778, 2.479, 3.172 and 0.618 e using WB97XD method. For the isolated ligand (**L**), the occupancy (energy) of the occupied donor atom natural orbital are 1.868 (-0.480 a.u) for LP(1)N, 1.980(-0.802 a.u) for LP1(O) and 1.859 (-0.329 au) for LP(2)O.
